# Sequencing, *De Novo* Assembly, and Annotation of the Transcriptome of the Endangered Freshwater Pearl Bivalve, *Cristaria plicata*, Provides Novel Insights into Functional Genes and Marker Discovery

**DOI:** 10.1371/journal.pone.0148622

**Published:** 2016-02-12

**Authors:** Bharat Bhusan Patnaik, Tae Hun Wang, Se Won Kang, Hee-Ju Hwang, So Young Park, Eun Bi Park, Jong Min Chung, Dae Kwon Song, Changmu Kim, Soonok Kim, Jun Sang Lee, Yeon Soo Han, Hong Seog Park, Yong Seok Lee

**Affiliations:** 1 Department of Life Science and Biotechnology, College of Natural Sciences, Soonchunhyang University, 22 Soonchunhyangro, Shinchang-myeon, Asan, Chungchungnam-do, 336-745, Republic of Korea; 2 National Institute of Biological Resources, Incheon, 404-170, Republic of Korea; 3 Institute of Environmental Research, Kangwon National University, 1 Kangwondaehak-gil, Chuncheon-si, Gangwon-do, 200-701, Republic of Korea; 4 College of Agriculture and Life Science, Chonnam National University, 300 Yongbong-Dong, Buk-gu, Gwangju, 500-757, Republic of Korea; 5 Research Institute, GnC BIO Co., LTD., 621-6 Banseok-dong, Yuseong-gu, Daejeon, 305-150, Republic of Korea; 6 Trident School of Biotech Sciences, Trident Academy of Creative Technology (TACT), Bhubaneswar- 751024, Odisha, India; CNRS UMR7622 & University Paris 6 Pierre-et-Marie-Curie, FRANCE

## Abstract

**Background:**

The freshwater mussel *Cristaria plicata* (Bivalvia: Eulamellibranchia: Unionidae), is an economically important species in molluscan aquaculture due to its use in pearl farming. The species have been listed as endangered in South Korea due to the loss of natural habitats caused by anthropogenic activities. The decreasing population and a lack of genomic information on the species is concerning for environmentalists and conservationists. In this study, we conducted a *de novo* transcriptome sequencing and annotation analysis of *C*. *plicata* using Illumina HiSeq 2500 next-generation sequencing (NGS) technology, the Trinity assembler, and bioinformatics databases to prepare a sustainable resource for the identification of candidate genes involved in immunity, defense, and reproduction.

**Results:**

The *C*. *plicata* transcriptome analysis included a total of 286,152,584 raw reads and 281,322,837 clean reads. The *de novo* assembly identified a total of 453,931 contigs and 374,794 non-redundant unigenes with average lengths of 731.2 and 737.1 bp, respectively. Furthermore, 100% coverage of *C*. *plicata* mitochondrial genes within two unigenes supported the quality of the assembler. In total, 84,274 unigenes showed homology to entries in at least one database, and 23,246 unigenes were allocated to one or more Gene Ontology (GO) terms. The most prominent GO biological process, cellular component, and molecular function categories (level 2) were cellular process, membrane, and binding, respectively. A total of 4,776 unigenes were mapped to 123 biological pathways in the KEGG database. Based on the GO terms and KEGG annotation, the unigenes were suggested to be involved in immunity, stress responses, sex-determination, and reproduction. A total of 17,251 cDNA simple sequence repeats (cSSRs) were identified from 61,141 unigenes (size of >1 kb) with the most abundant being dinucleotide repeats.

**Conclusions:**

This dataset represents the first transcriptome analysis of the endangered mollusc, *C*. *plicata*. The transcriptome provides a comprehensive sequence resource for the conservation of genetic information in this species and enrichment of the genetic database. The development of molecular markers will assist in the genetic improvement of *C*. *plicata*.

## Introduction

*Cristaria plicata* (Leach, 1815), a well-known “freshwater pearl bivalve”, belongs to the order Unionoida and family Unionidae under the phylum Mollusca. The species has restricted geographic distribution in Russia, Japan, Vietnam, Laos Republic, Thailand, Cambodia, and a wider presence in China, where it is used for freshwater pearl farming, medicinal purposes, and as a model for aquaculture industries [1–3]. In South Korea, *C*. *plicata* is found in the middle and lower sections of the Nakdong River, in Asan Lake in Chungcheongnam-do, and in Gosean Lake in Chungcheogbuk-do. The species has been classified as vulnerable owing to loss of natural habitats caused by river development, reduced host fish populations, and indiscriminate collection. Due to a rapid decrease in its population in recent years, *C*. *plicata* has been listed in the Korean Red List of Threatened Species under the endangered wildlife category by the Ministry of Environment and is protected by law. Under the International Union for Conservation of Nature and Natural Resources (IUCN) Red List of Threatened species, *C*. *plicata* has been assessed as data deficient with indications of localized decreases in the population [4].

Due to limited sample resources and genomic information, an exhaustive survey of novel candidate genes involved in local adaptation, the immune system, and reproduction for *C*. *plicata* is absent. The complete mitochondrial genome sequence and functional analysis of a few oxidative stress and immunity- related genes are the only available reports of *C*. *plicata* genetic information [5–9]. Although the available information increases our understanding of the phylogeny and molecular basis of the innate immune response in *C*. *plicata*, it is insufficient to address the sustainability and conservation of the species. To identify strategies for the local adaptation of the species, knowledge of the genes and pathways involved in the immune system and reproduction are required. *C*. *plicata* molecular markers, which are required for marker-assisted selection programs in aquaculture, remain poorly explored. The discovery of molecular markers generally acts as a catalyst for the study of genetic diversity and population structure. The identification of novel genomic resources using a rapid and cost-efficient approach for the conservation of *C*. *plicata* in its natural habitat is important.

High-throughput next-generation sequencing (NGS) technologies, with their improved efficiency, cost benefits, and rapid data production, have been useful for understanding the mechanisms underlying the diversity of non-model organisms including American bullfrog (*Rana catesbeiana)* [10], polychaetes (*Hermodice carunculata)* [11], amphipods (*Melita plumulosa)* [12], green odorous frog (*Odorana margaretae)* [13], fish (*Salmo salar)* [14], shrimp (*Litopenaeus vannamei)* [15], and giant freshwater prawn (*Macrobrachium rosenbergii)* [16]. The Solexa/Illumina and 454/Roche NGS technologies have been revolutionary for understanding the rich transcriptomes of the molluscs. Many sequencing projects involving molluscs have used the Roche 454 Genome Sequencing FLX technology due to its faster production of accurate datasets. These have included transcriptome datasets for a bivalve mussel (*Limnoperna fortunei)* [17], small abalone (*Haliotis diversicolor)* [18], blue mussel (*Mytilus edulis)* [19], Manila clam (*Ruditapes philippinarum)* [20], Yesso scallop (*Patinopecten yessoensis)* [21], and an Antarctic bivalve (*Laternula elliptica)* [22], among others. Illumina sequencing technology, which is more efficient, provides shorter reads, and provides greater coverage, has been used for many molluscan transcriptome sequencing projects including blood cockle (*Anadara trapezia)* [23], Japanese scallop (*Mizuhopecten yessoensis)* [24], Eastern Oyster (*Crassostrea virginica)* [25], South African abalone (*Haliotis midae)* [26], and a snail species (*Radix balthica)* [27]. Furthermore, in a mollusc phylogenomics study, the matrix completeness of Illumina data was shown to be superior to that of 454 data [28]. Advances in assembly algorithms and relatively inexpensive work-flow have made Illumina sequencing the preferred choice with respect to transcriptome studies of endangered species [29,30].

*De novo* transcriptome analysis of *C*. *plicata* using Illumina short read sequencing and annotation of a high-quality transcriptome assembly can be used to increase our understanding of the diversity of genes. *C*. *plicata* is an endangered species and new genomic resources may serve as an important public information platform for conservation of the species in Korea and in progressive pearl culture production in the farming communities of other countries. Our transcriptome dataset provides the first characterization of expressed sequences in the pearl mollusc, *C*. *plicata*, including the identification of candidate genes involved in immunity and reproduction. Furthermore, simple sequence repeats (SSRs) generated from the transcriptome data may be useful for genetic improvement of this species.

## Materials and Methods

### Ethics statement

This study has been accorded permission (Ref. No. 2014–10) from the Guem River Basin Environmental Office.

### Biological samples and RNA extraction

A single *C*. *plicata* specimen was used for RNA sequencing in this study due to restricted use of the species for experimental purposes as declared by the Ministry of Environment, South Korea. The specimen was collected from Sapgyoho Lake, Asan-si, Chungnam, South Korea. After transferring the specimen to the laboratory, the visceral pouch tissue was dissected and immediately placed into liquid nitrogen until RNA preparation. For RNA extraction, the snap-frozen *C*. *plicata* visceral mass tissue was homogenized using Trizol Reagent (Invitrogen) according to the manufacturer’s instructions. The purity and integrity of RNA preparations were determined using a NanoDrop-2000 spectrophotometer (Thermo, USA) and a Bioanalyzer 2100 (Agilent Technologies, USA).

### Construction of the mRNA-seq library and Illumina Sequencing

An mRNA-seq library was constructed using the mRNA-seq sample preparation kit (Illumina, San Diego, CA) following the manufacturer’s instructions. Briefly, poly (A)^+^ mRNA was purified from total RNA samples with oligo(dT) magnetic beads and fragmented using an RNA fragmentation kit (Ambion, Austin, TX) prior to cDNA synthesis. The short mRNA fragments were reverse-transcribed into first-strand cDNA using reverse-transcriptase (Invitrogen, Carlsbad, CA) and random hexamer-primers. Second-strand cDNA synthesis was accomplished using DNA polymerase I (New England BioLabs, Ipswich, MA) and RNase H (Invitrogen). The double-stranded cDNA was end-repaired using T4 DNA polymerase (New England BioLabs), the Klenow fragment (New England BioLabs), and T4 polynucleotide kinase (New England BioLabs). The end-repaired cDNA fragments were ligated with PE Adapter Oligo Mix using T4 DNA ligase (New England BioLabs) at room temperature for 15 min. The ligated products were purified and separated by size on an agarose gel. DNA fragments of the desired size (200 ± 25 bp) were excised and, after validation, were sequenced on the Illumina HiSeq 2500 sequencing platform.

### *De novo* assembly

Before *de novo* transcriptome assembly, the raw reads were cleaned by removing adaptor-only reads (recognized adaptor length ≤ 13 nucleotides and remaining adaptor-excluded length ≤ 35 nucleotides), repeated reads, and low-quality reads (Phred quality score < 20) using the Sickle software tool (http://github.com/najoshi/sickle) [31] and Fastq_filter software (part of the Galaxy toolshed) [32]. The remaining high-quality reads were assembled using the short read assembling program Trinity with 100 GB of memory and a path reinforcement distance of 50 [33]. The Trinity program (the default options and a minimum allowed length of 200 bp) first assembles reads of a certain length that overlap to form longer fragments without gaps called contigs. The total number of contigs and the mean length, N_50_ length, and GC% were recorded for the *de novo* assembly. These contigs were connected using the sequence clustering software TGICL [34] to obtain sequences that could no longer be extended on either end. Such sequences were defined as unigenes. They represent expressed assembled sequences, but are not characterized sufficiently to be represented as a gene.

### Transcriptome annotation and discovery

For the annotation profile of *C*. *plicata* unigenes, we first constructed a unique reference dataset that combined protein sequence data of Arthropoda, Nematoda, and Mollusca downloaded from the Taxonomy browser of the NCBI nr database. The sequences were converted to multi-FASTA format and stored in the PANM reference database (PANM DB) [35] using the formatdb program (downloaded from ftp://ftp.ncbi.nlm.nih.gov/blast/executables/release/2.2.26/). PANM DB is freely downloadable from the amino acid database BLAST web-interface of the Malacological Society of Korea (http://malacol.or.kr/blast/aminoacid.html). The assembled *C*. *plicata* unigene sequences were searched against the PANM DB reference database using the BLASTx algorithm [36] with an E-value threshold of 1.0E -5 to identify putative functional mRNA transcripts. Subsequently, the BLASTx hits of the assembled sequences against the UniGene DB were also recorded. The BLAST2GO software suite [37] was used to predict Gene Ontology (GO) terms [38], assign the assembled sequences to the Kyoto Encyclopedia of Genes and Genomes (KEGG) pathways [39], and to identify protein domains against the InterPro databases using the InterProScan tool [40]. Annotations using BLAST2GO were conducted with 1.0E -6 as the E-value hit filter, 55 as the annotation cut-off and 5 as the GO weight. No HSP-hit coverage cut-off was considered. The GO terms were classified into three categories, hence, we generated separate graphs (pie chart at level 2) for biological process, cellular component, and molecular functions. The unigenes were also predicted by a query of the NCBI Clusters of Orthologous Groups (COG) DB (BLASTx, E-value cutoff of 1E -5) [41].

### Identification of candidate genes related to immune responses and reproduction

Identification of candidate *C*. *plicata* genes involved in immune responses, sex-determination, and reproduction was performed using a keyword search of our BLASTx annotation results in the PANM DB. A set of keywords, composed of a series of representative innate immunity and oxidative stress genes, was used to predict immune response genes based on annotation results. Similarly, representative sex-determination and sex-differentiation genes were used to search for reproduction- related unigenes from the annotation results. In addition, the GO terms and KEGG classification information were required to identify important candidate genes. GO terms such as “immune system process”, “response to stimulus”, “signaling” under the biological process domain and “anti-oxidant activity” under the molecular function domain were used to scan for the immune response genes. Similarly, the GO terms “reproduction” and “reproductive process” were used to select candidate genes involved in reproduction of the mollusc. In addition, the KEGG category “immune system” was also used to identify candidate immune response genes.

### Microsatellite marker discovery

The assembled *C*. *plicata* unigenes were searched for simple sequence repeat (SSR) motifs using the MIcroSAtellite (MISA) software [42]. For SSR identification only >1 kb sequences of unigenes were considered. All types of SSRs, from dinucleotide to hexanucleotide repeats were examined. The searches were run with the minimum repeat number of six for dinucleotide repeats and five for all other repeat motifs.

## Results and Discussion

### Illumina Hi-Seq 2500 sequencing and assembly evaluation

**Fig 1:**
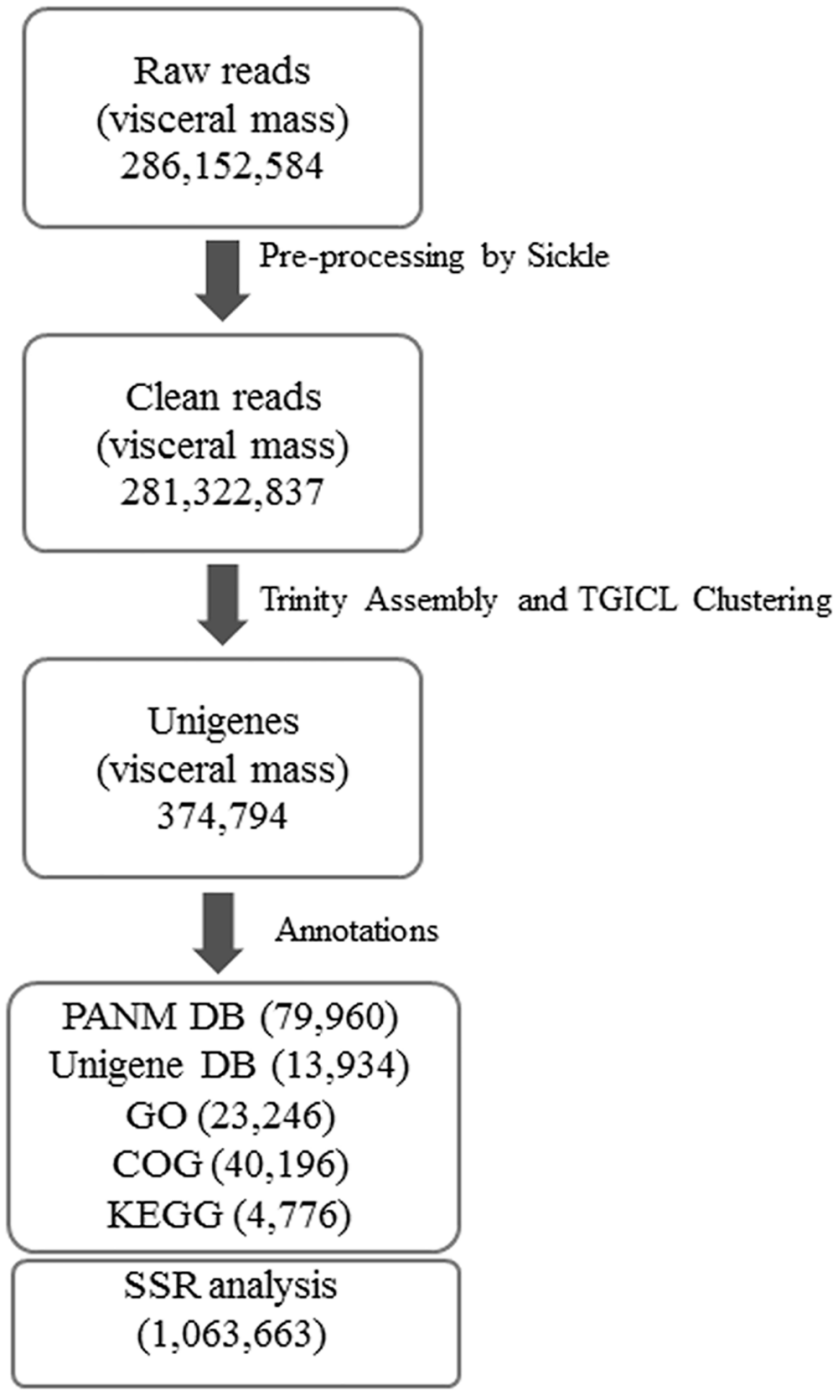
Schematic representation of transcriptome assembly and annotation. A *C*. *plicata* visceral mass transcriptome was obtained using an Illumina HiSeq2500 NGS platform. The raw reads obtained were preprocessed using the Sickle software tool (quality: 20, length: 40) and Fastq_filter software to obtain clean reads. Trinity assembly (K-mer, 25; minimum contig length; 200) and TGICL clustering (Identity; 94%; overlap; 30 bp) generated 374,794 unigenes. The unigenes were used for functional annotation using the PANM, Unigene, COG, GO, and KEGG databases and structural annotation for SSR detection.

Transcriptome information for the endangered freshwater mollusc, *C*. *plicata*, was characterized by constructing a cDNA library prepared from purified mRNA isolated from the visceral mass tissue. The Illumina Hi-Seq 2500 sequencing platform generated a total of 286,152,584 raw reads with 36,055,225,584 bases. The raw reads were filtered to remove adaptor sequences, low quality reads (reads with more than 50% of bases having a Q-value ≤ 20), and ambiguous bases. A total of 281,322,837 clean reads (Phred quality ≥ Q20) with an average length of 124.1 bases was obtained. The transcriptome sequencing, assembly, and annotation scheme used for *C*. *plicata* are depicted in Fig 1. The clean reads obtained using the Illumina Hi-Seq 2500 transcriptome sequencing platform constituted 98.31% of the raw reads. The *C*. *plicata* transcriptome sequencing and assembly statistics are shown in Table 1. The Illumina sequence data for *C*. *plicata* were deposited in the NCBI Sequence Read Archive (SRA) under accession number SRP062467. The raw, untrimmed Illumina HiSeq2500 data along with the transcriptome assembly have been included as NCBI BioProject PRJNA293023.

**Table 1:** Transcriptome assembly statistics of *C*. *plicata* visceral mass using the Trinity analysis.

Description	Statistics
**Total number of raw read sequences**	**286,152,584**
Number of bases	36,055,225,584
Mean length (bp)	126
**Total number of clean read sequences**	**281,322,837**
Number of bases	34,909,374,303
Mean length (bp)	124.1
N50 length	126
High quality reads (%)	98.31 (sequences), 96.82 (bases)
**Total number of contigs**	**453,931**
Number of bases	331,930,879
Mean length of contig (bp)	731.2
N50 length of contig (bp)	1,254
GC% of contig	36.62
Largest contig (bp)	36,440
No. of large contigs (≥500 bp)	151,695
**Total number of unigenes**	**374,794**
Number of bases	276,264,683
Mean length of unigene (bp)	737.1
N50 length of unigene (bp)	1,262
GC% of unigene	36.47
Length ranges (bp)	212–68,788

**Fig 2:**
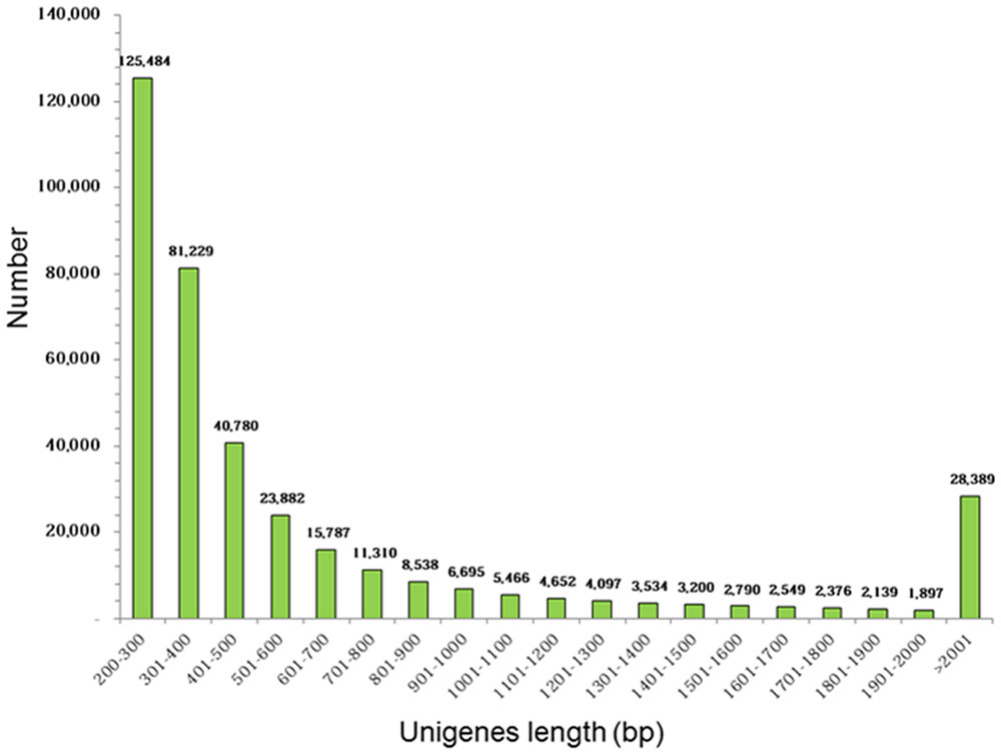
Summary of *C*. *plicata* visceral mass unigene (≥ 200 bp) sequences after Trinity assembly.

High-quality reads generated from the transcriptome sequencing of the *C*. *plicata* visceral mass tissue were subsequently assembled using the Trinity program because assembled and annotated genomic sequence information for the *Cristaria* species were not available. The Trinity *de novo* assembler is used for the assembly of trimmed reads with an optimal K-mer length of 25. Trinity is the first program designed specifically for *de novo* transcriptome assembly and utilizes a novel method for the reconstruction of transcriptomes from RNA-seq data using three sequential software modules; namely, Inchworm, Chrysalis, and Butterfly [43]. Other short-read transcriptome assemblers such as Oases [44], Trans-ABySS [45], SOAPdenovo-Trans [46], and Rnnotator [47] are available, which are essentially modifications from the genome assembly. The Trinity assembler generated a total of 453,931 contigs with 331,930,879 bases (N_50_ length, 1,254 bp; mean length, 731.2 bp) and a GC% of 36.62%. The contigs were assembled into a total of 374,794 unigenes with 276,264,683 bases. The N_50_ length and the mean length of unigenes produced were 1,262 and 737.1 bp, respectively, with a GC% of 36.47. The lengths of the smallest to largest unigenes in the *C*. *plicata* transcriptome ranged from 212–68,788 bp. Among these unigenes, 125,484 unigenes (33.48%) were no more than 300 bp, 188,221 unigenes (50.22%) were 301–1,000 bp, 32,700 (8.72%) unigenes were 1,001–2,000 bp, and 28,389 unigenes (7.57%) were greater than 2,000 bp (Fig 2).

Complete coverage of the *C*. *plicata* mitochondrial transcriptome was demonstrated with the Trinity assembler (S1 Table). The 100% sequence coverage of the 13 protein-coding genes of the *C*. *plicata* mitochondrial genome using only two assembled unigenes (unigene Cp_000887 and unigene Cp_009974) demonstrated the integrity and completeness of Trinity *de novo* assembler. Several assemblers have been tested to map mitochondrial protein-coding genes in assembled contig sequences with a lesser degree of coverage [25,27]. The coverage of mitochondrial DNA genes is a direct measure of the quality of assembled sequences. An earlier study reported that the Oases analysis pipeline with a K-mer size of 23 was the best program for assembling the *de novo* transcriptome of *Crassostrea virginica* compared to the SOAPdenovo-Trans (K-mer sizes of 41 and 51) and Trinity (K-mer size of 25) programs based on the N_50_ length of contigs, the number of contigs longer than 500 bp, and the alignment coverage [25]. The Trinity program used for the transcriptome assembly of *C*. *plicata* RNA-seq reads with average contig and unigene lengths of 731.2 bp and 737.1 bp, respectively, was found better than or similar to most other Illumina sequenced assemblies: 260 and 434bp average contig lengths in *H*. *midae* [26] and *R*. *balthica* [27], respectively, and 706 and 580bp average unigene lengths in the endangered species Chinese sturgeon, (*Acipenser sinensis)* [30] and Chinese salamander (*Hynobius chinensis)* [29], respectively. A comprehensive summary of molluscan transcriptomes in the last three years using NGS platforms (Table 2) shows a preference for Illumina technology combined with the Trinity assembly process. Transcriptome data on endangered or endemic molluscs obtained using NGS platforms would increase our understanding of their genomic attributes and provide information for species conservation in their natural environment. Thus, the *C*. *plicata* transcriptome and annotation of valuable genes will be useful for functional genomics research and the development of molecular markers, and will serve as reference information for closely related species.

**Table 2:** Summary of molluscan transcriptomics in the last three years using Next Generation Sequencing (NGS) platforms^#^. R- raw reads, C- clean reads;

Species (Tissue)	NGS platform	Reads (n)	Assembler	Contigs (n)	Contigs (mean length in bp)	Contigs (N50 length in bp)	Others	SRA Accession	Sequencing objectives	Reference
*Cristaria plicata*	Illumina HiSeq2500	R- 286,152,584 C- 281,322,837	Trinity	453,931	731.2	1,254	374,794^b^	SRP062467 PRJNA293023	Endangered species	This study
*Crassostrea hongkongensis*	Roche 454GS FLX	R- 1,595,855 C- 1,405,240	GS Denovo Assembler v2.6	41,472	958	1,571	—	SRR949615	Genetic selection	[48]
*Tritonia diomedea* (Central Nervous System)	Illumina HiSeq2000	R- 133,156,930	Trinity	185,546	74	363	—	PRJNA252890	Molecular correlates of behavior	[49]
*Octopus vulgaris* (hemocytes)	Illumina GAIIx	R- 150,302,926 C- 127,019,711	Trinity	254,506	669	1,632	87,408^a^	SRP043705	Molecular basis of immune defense	[50]
*Mytilus galloprovincialis* (Digestive gland)	Illumina	R- 57,059,700 C- 52,770,704	Trinity	21,193	771	1,010	—	SRP011280.2	Molecular markers for toxin accumulation	[51]
*Perna viridis* (adductor muscle, gills, hepatopancreas)	Illumina Genome Analyzer IIx	C- 544,272,542	Trinity	233,257	1,264	2,868	—	SRP043984	Molecular aspects of toxicity responses	[52]
*Anadara trapezia* (Multiple tissue types)	Illumina HiSeq2000	C- 27,000,000	Trinity	75,024	505	597	13,507	PRJNA210944	Physiological response to environment stress	[23]
*Pinctada maxima* (mantle)	Illumina HiSeq2000	R- 49,500,748	Trinity	108,704^b^	407	—	—	—	Development of EST-SSR markers	[53]
*Echinolittorina malaccana*	Illumina HiSeq2000	R- 61,000,000	Trinity	115,211^b^	453	492	—	SRP041635	Thermal adaptations	[54]
*Pecten maximus* (mantle)	Illumina HiSeq2000	R- 1,335,123,074	SOAPdenovo	26,064	1,011	—	—	SRP040427	Shell production	[55]
*Pecten maximus* (hemocyte)	Illumina HiSeq2000	R- 216,444,674	CLC Genomic workbench	73,752	502.6	—	—	SRR1009240, SRR1009241, SRR1009242	Immunity	[56]
*Corbicula fluminea* (Multiple tissue types)	Illumina GAIIx	R- 67,087,130 C- 62,250,336	Velvet & Oasis	134,684^b^	791.06	1,264	—	SRA062349	Exploration as environmental test organism	[57]
*Chlamys farreri* (mantle)	Illumina HiSeq2000	R- 59,918,916 C- 55,122,820	Trinity	188,629	249	306	—	—	Shell production	[58]
*Sinonovacula constricta* (Multiple tissue types)	Roche 454GS FLX	C- 859,313	Newbler2.7	16,323	1,376	—	—	GALB01000000	Molecular markers for growth	[59]
*Mizuhopecten yessoensis* (Multiple tissue types)	Illumina GAIIx	R- 112,265,296	Velvet & Oases	217,190	436	—	—	SRR653778	Molecular response to heavy metals	[24]

^a^ number of contigs no less than 500 bp;

^b^ number of unigenes

^#^ For a summary of molluscan transcriptome analysis prior to 2013, please refer [25]

### Sequence annotation of unigenes

The assembled unigenes in the *C*. *plicata* transcriptome were used to conduct a BLASTx search (E-value ≤ 1E-5) against the curated PANM DB, UniGene DB, and COG DB for validation and annotation of genes. Of 374,794 unigenes, 79,960 (21.33%), 40,196 (10.72%), and 13,934 (3.72%) unigenes were similar to sequences in the PANM, COG, and UniGene DBs, respectively (Table 3). The majority of unigenes annotated to homologous sequences in the DBs had lengths ≥1000 bp. A total of 39,682 (10.59%) and 11,368 (3.03%) unigenes had common homologous matches in the PANM with COG DBs, and the PANM with UniGene DBs, respectively. A total of 9,820 (2.62%) unigenes were annotated simultaneously by all three DBs. In total, 84,274 (22.49%) annotations were found within the clustered unigenes of the *C*. *plicata* transcriptome. The non-annotated unigene sequences were less likely to produce BLAST hits in the protein databases possibly due to their shorter sequences and their lack of a representative protein domain.

**Table 3:** Functional annotation of unigenes of the *Cristaria plicata* transcriptome.

Databases	All annotated transcripts	≤300 bp	300–1000 bp	≥1000 bp
PANM	79,960	14,480	30,748	34,732
UniGene	13,934	1,848	3,721	8,365
COG	40,196	4,763	11,445	23,988
GO	23,246	2,593	5,625	15,028
KEGG	4,776	483	927	3,366
All annotated	84,274	15,700	33,108	35,466

### Homology characteristics and functional annotation of unigenes

**Fig 3:**
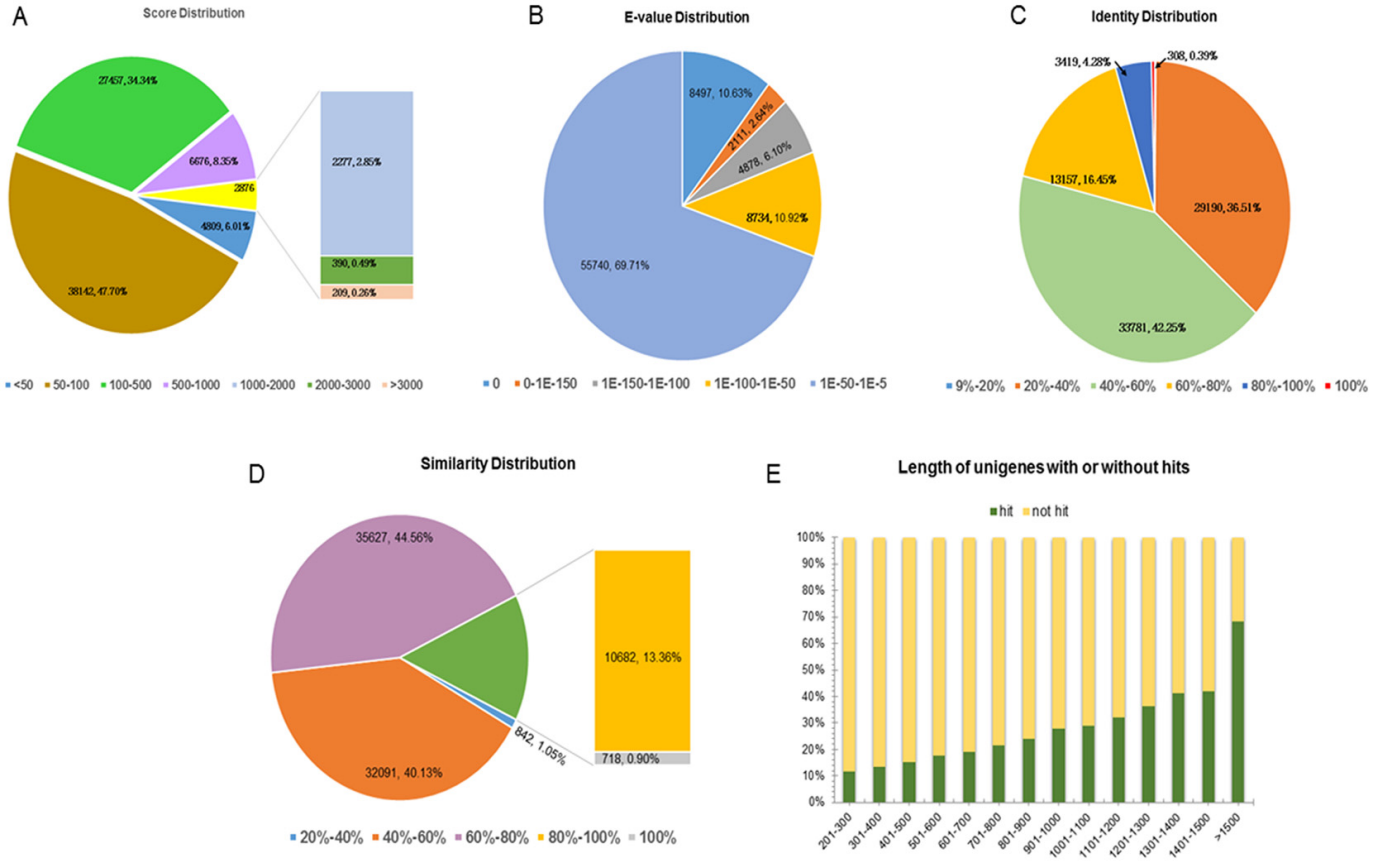
Statistical summary of homology search of assembled unigenes against the PANM protein database. **(A)** Score distribution of BLAST hits for each unigene with a cutoff E-value of 1E -5. **(B)** E-value distribution of each unigene using BLAST hits with a cutoff E-value of 1E -5. **(C)** Identity distribution of the top BLAST hits for each unigene. **(D)** Similarity distribution of the top BLAST hits for each unigene. **(E)** Lengths of unigenes compared with the presence or absence of BLAST hits.

**Fig 4:**
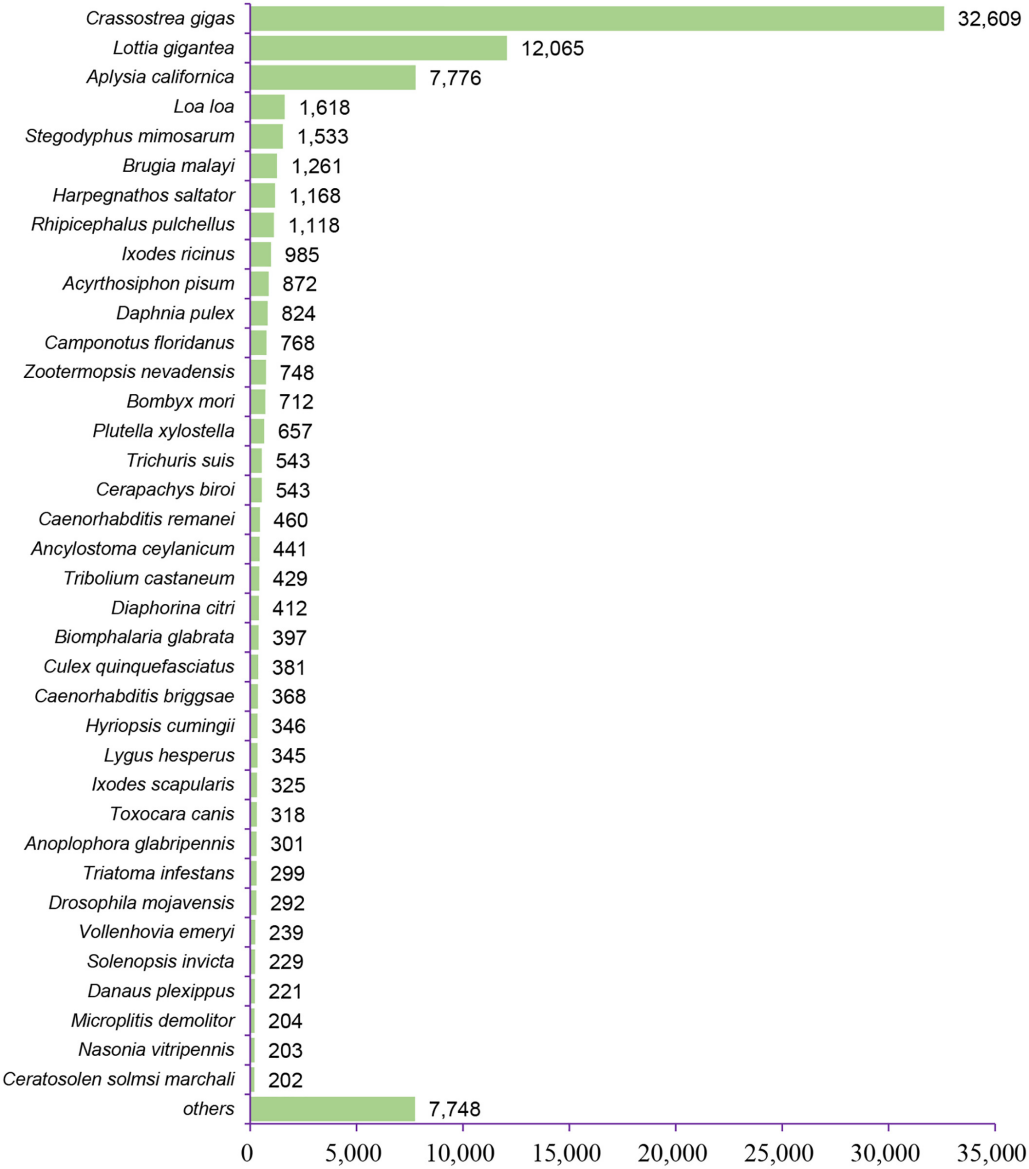
Top-hit species distribution of *C*. *plicata* visceral mass unigenes against the PANM database (custom-devised curatable database of mollusc, arthropod, and nematode protein sequences downloaded from the NCBI nr database). An E-value cutoff of 1E -5 was maintained and the hit distribution shows high homology to known genome sequences of the Mollusca phylum.

Characteristics of the homology search of assembled unigenes against the PANM DB are summarized in Fig 3. The score distribution, which represents the quality of the BLAST alignment, showed that 38,142 (47.70%) unigenes had scores between 50 and 100 and 27,457 unigenes had scores between 100 and 500 (Fig 3A). Only 4,809 (6.01%) unigenes had a score < 50 reflecting the quality of the assembly and sequence annotation process. The E-value distribution revealed that 55,740 unigenes (69.71%) showed significant homology to deposited sequences (1E- 50 to 1E- 5, Fig 3B). The identity distribution showed that 33,781 (42.25%) and 16,884 (21.12%) unigenes showed identities of 40–60% and > 60%, respectively, to deposited sequences (Fig 3C). In addition, the similarity distribution showed that 47,027 (58.81%) unigenes had similarities greater than 60% (Fig 3D). The lengths of unigenes were directly related to the presence or absence of BLAST hits (Fig 3E). This is understandable since the longer sequences are more likely to contain protein domain characteristics and are more likely to have BLAST matches in the protein database. Another basis for understanding unigene characteristics is the BLAST top-hit species distribution which shows putative homology of the annotated sequence across species in the PANM DB (Fig 4). Based on our analysis, the highest homology was observed with the oyster, *Crassostrea gigas* (32,609 unigenes, 40.78%), followed by the owl limpet, *Lottia gigantea* (12,065 unigenes, 15.09%). As expected, the majority of unigene hits belonged to molluscan and other arthropod proteins. A summary of the top-hit InterPro domains identified 1,374 unigenes with zinc finger, C2H2-like domains. Zinc finger domains participate in important cell processing functions including signal transduction and transcriptional regulation and are a common feature in molluscs, insects, and other crustacean groups [60,61]. The C2H2-like zinc finger proteins are the most common DNA-binding motifs present in prokaryotic and eukaryotic transcription factors [62]. Other top domains identified based on unigene homology included the Toll/interleukin-1 receptor homolog (TIR) domain, C-type lectin domain, death-like domain, and heat shock protein 70 family domain, which are putative candidates for involvement in immune signaling processes in *C*. *plicata*. The 40 top-hit InterPro domains in the *C*. *plicata* transcriptome are summarized in Table 4.

**Table 4:** List of the top-hit 40 InterPro domains in *C*. *plicata* transcriptome.

InterPro domain	Description	Unigenes
IPR015880	Zinc finger, C2H2-like domain	1374
IPR027417	P-loop containing nucleoside triphosphate hydrolase domain	1126
IPR000477	Reverse transcriptase domain	637
IPR012337	Ribonuclease H-like domain	496
IPR011042	Six-bladed beta-propeller, TolB-like domain	491
IPR013783	Immunoglobulin-like fold domain	465
IPR001841	Zinc finger, RING-type domain	405
IPR002110	Ankyrin repeat	404
IPR005135	Endonuclease/Exonuclease/phosphatase domain	388
IPR000315	B-box-type zinc finger domain	350
IPR000276	G protein-coupled receptor, rhodopsin-like family	320
IPR002290	Serine/threonine/dual specificity protein kinase, catalytic domain	272
IPR001370	BIR repeat	229
IPR003599	Immunoglobulin subtype domain	223
IPR024810	Mab-21 domain	219
IPR000504	RNA recognition motif domain	214
IPR000242	PTP type protein phosphatase domain	214
IPR002035	von Willebrand factor, type A domain	213
IPR003615	HNH nuclease domain	211
IPR027124	SWR1-complex protein 5/Craniofacial development protein family	209
IPR002048	EF-hand domain	209
IPR013083	Zinc finger, RING/FYVE/PHD-type domain	196
IPR000742	EGF-like domain	195
IPR008979	Toll/interleukin-1 receptor homology (TIR) domain	193
IPR000157	Galactose-binding domain-like	193
IPR002126	Cadherin domain	189
IPR003591	Leucine-rich repeat, typical subtype repeat	179
IPR000436	Sushi/SCR/CCP domain	175
IPR001680	WD40 repeat	173
IPR003593	AAA+ ATPase domain	172
IPR001304	C-type lectin domain	169
IPR011029	Death-like domain	163
IPR013087	Zinc finger C2H2-type/integrase DNA-binding domain	159
IPR011701	Major facilitator superfamily	158
IPR015943	WD40/YVTN repeat-like-containing domain	150
IPR001128	Cytochrome P450 family	150
IPR019734	Tetratricopeptide repeat	145
IPR013126	Heat shock protein 70 family	145
IPR000210	BTB/POZ domain	136

**Fig 5:**
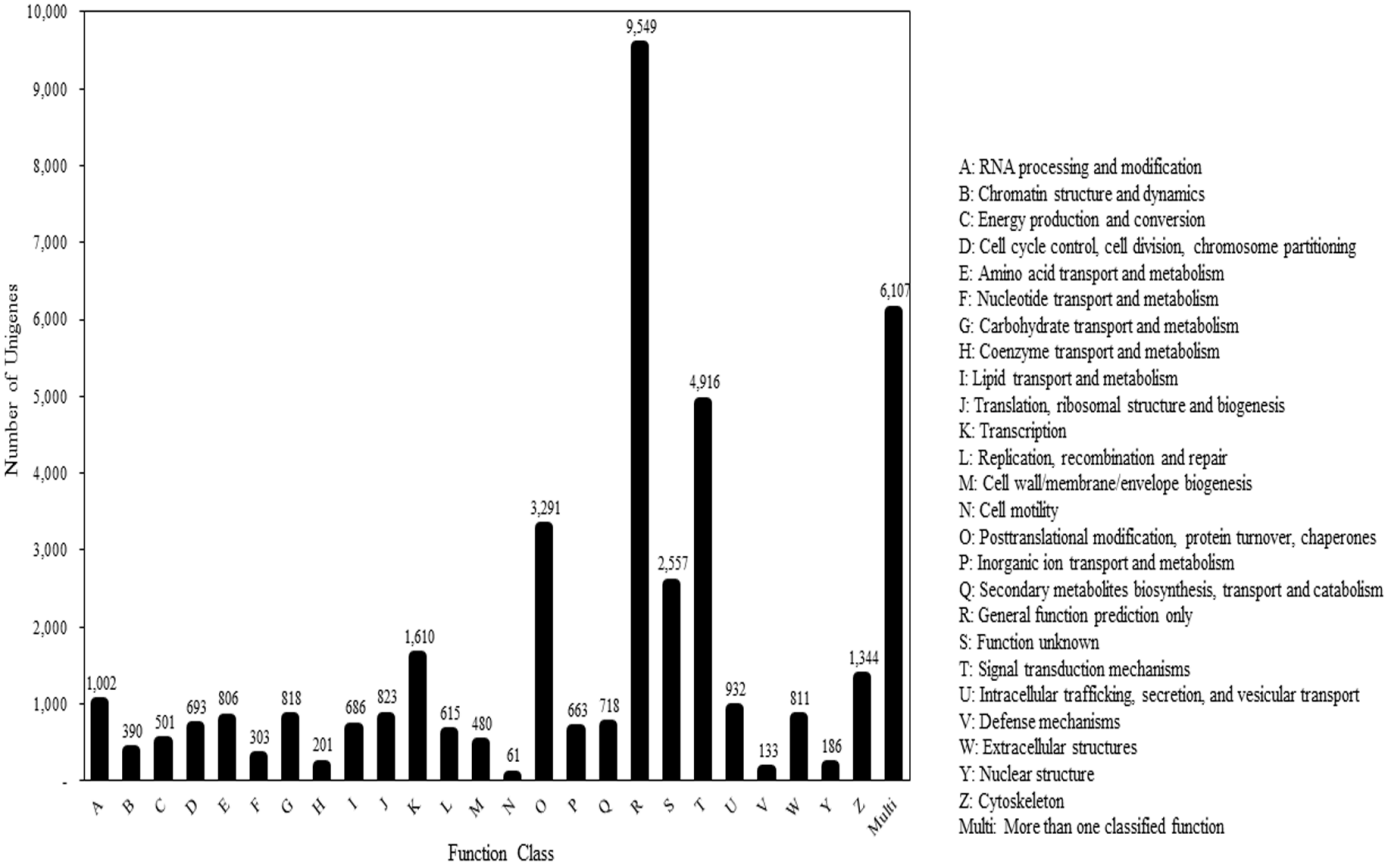
Clusters of orthologous groups (COG) classification of unigenes. Out of 374,794 annotated unigenes, 40,196 sequences had a COG classification from among the 25 COG categories (excluding the multi category).

We subjected all unigenes to a search against the COG DB to make functional predictions. The unigenes were distributed among 25 functionally classified categories (excluding the multi category) (Fig 5). Among the 25 COG categories, the “general function prediction” cluster constituted the largest group (9,549; 23.75%), followed by “signal transduction mechanisms” (4,916; 12.23%), “post-translational modification, protein turnover, chaperones” (3,291; 8.19%), “function unknown” (2,557; 6.4%), “transcription” (1,610; 4%), “cytoskeleton” 1,344; 3.3%), and “RNA processing and modification” (1,002; 2.5%). A greater number of unigenes (6,107; 15.19%) were also allocated to the multi assignment category.

**Fig 6:**
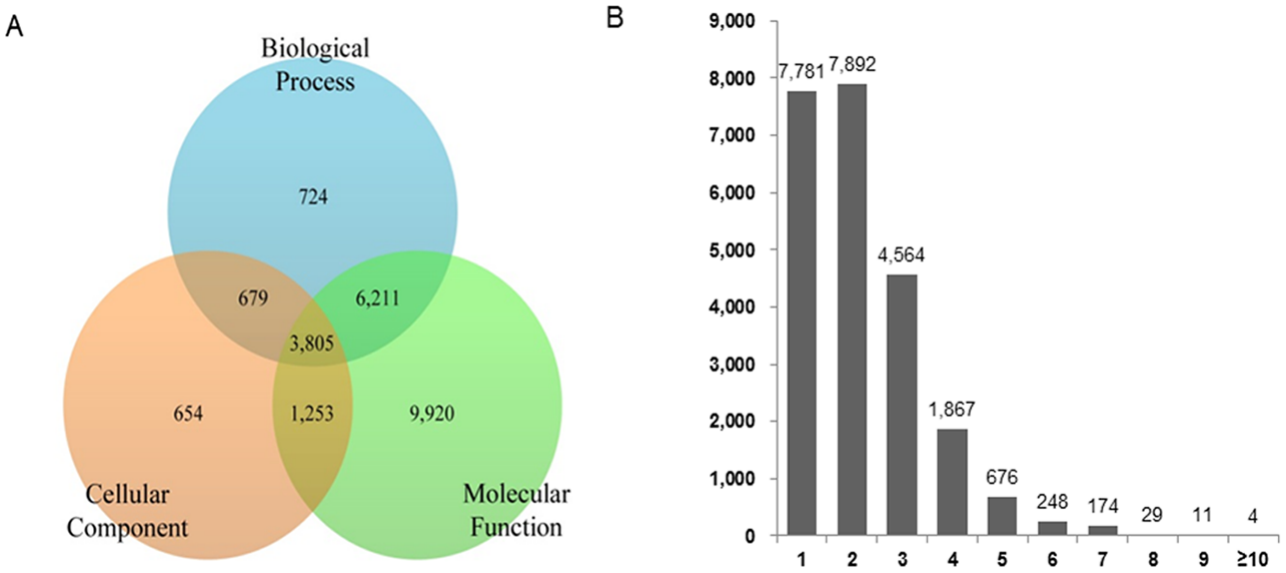
Functional annotation of *C*. *plicata* visceral mass assembled sequences based on gene ontology (GO) categorization. **(A)** An overlap model of the annotated unigenes assigned to biological processes, molecular functions, and cellular components based on GO function. **(B)** Numbers of unigenes assigned to GO term annotations.

Gene Ontology (GO)-based annotation is an internationally standardized gene functional classification system that describes gene products in terms of their associated biological processes, cellular components, and molecular functions. To make functional predictions for the *C*. *plicata* unigenes, we mapped the associated GO terms to 79,960 unigenes that had BLAST matches. The GO annotation was based on the BLASTx results against the nr database. Protein domains and motif information were retrieved using the InterProScan sequence search tool via BLAST2GO and the annotation was merged with already existing GO terms. After merging, the 23,246 unigenes (11,419 for biological process, 6,391 for cellular component, and 21,189 for molecular function) were assigned one or more GO terms based on sequence similarity. Furthermore, 10,016 (43.09% of the 23,246 unigenes) were annotated with both biological processes and cellular components, 5,058 (21.76%) were annotated with both cellular components and molecular function, 4,484 (19.29%), annotated with both biological process and cellular components, while 3,805 (16.37%) were assigned to all three categories (Fig 6A). A calculation of the number of unigenes associated with GO terms suggested that 7,892 and 7,781 sequences were associated with two or one GO term annotations, respectively (Fig 6B). As expected, the evidence code distribution showed an over-representation of electronic annotations that have not been created manually and may contain higher false positives. The evidence code ‘inferred from electronic annotation’ (IEA) like others, such as ‘inferred from sequence or structural similarity’ (ISS), ‘inferred from reviewed computational analysis’ (RCA), and ‘inferred from genomic context’ (IGC) belong to computational source of evidence; those that constitute over 95% of the total GO annotation analysis [63]. Hence, with respect to our GO term annotation results, all of the GO terms are not of equal validity and, based on this, the interpretation of unigenes relates to only the predicted function.

**Fig 7:**
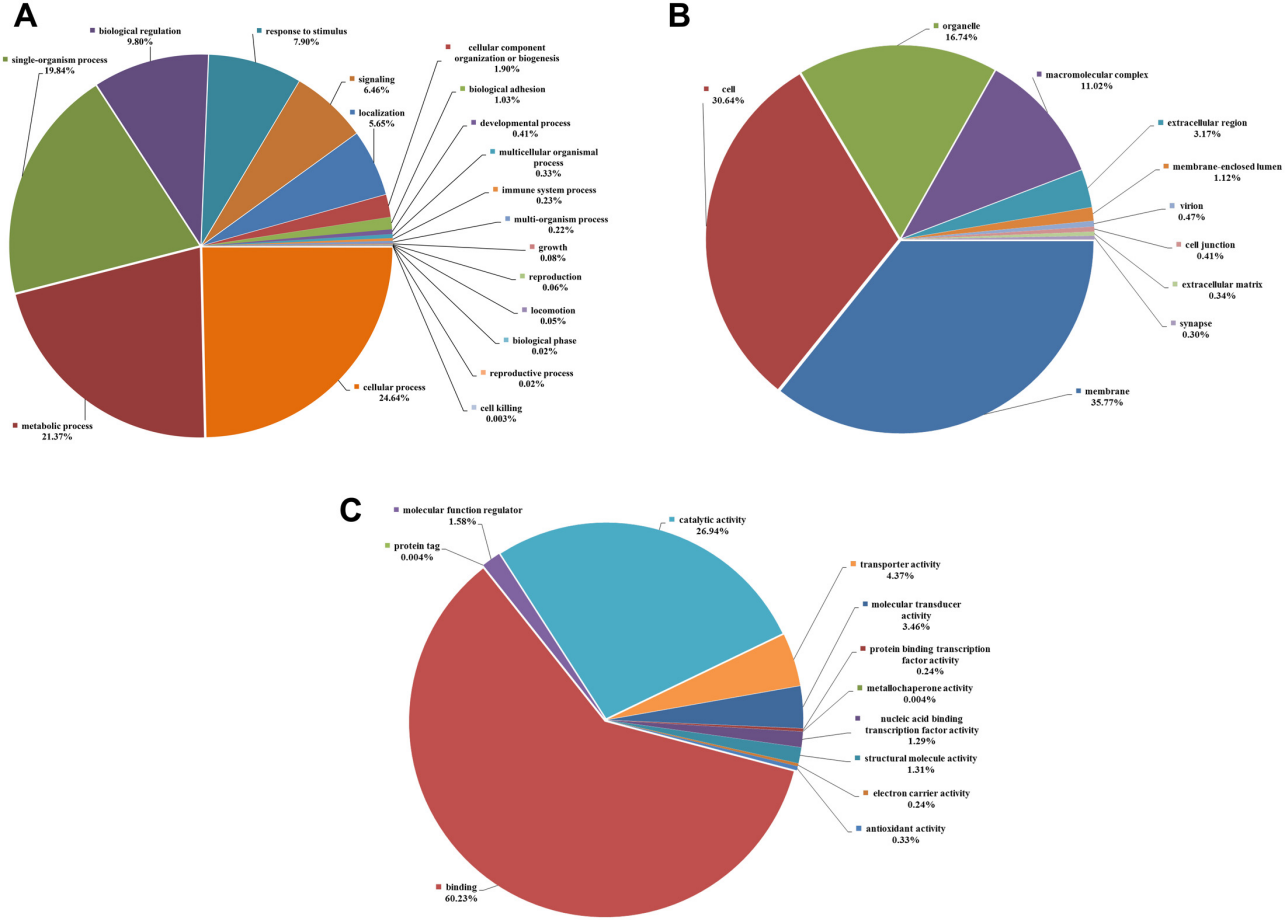
GO classifications of the *C*. *plicata* transcriptome at level 2. GO analyses were performed for three major classification categories: **(A)** biological processes; **(B)** cellular components and **(C)** molecular functions.

Among the 23,246 unigenes for which we obtained GO terms, we observed a wide diversity of functional categories represented on level 2 of the GO database. Fig 7 shows a total of 19 biological process, 10 cellular components, and 12 molecular function GO level-2 classes in which the unigenes were predicted to function. Within the biological processes category, genes involved in cellular processes (GO:0009987) and metabolic processes (GO:0008152) were represented prominently, followed by single-organism processes (GO:0044699) and biological regulation (GO:0065007) (Fig 7A). In the cellular component category, membrane (GO:0016020), cell (GO:0005623), and organelle (GO:0043226) represented the majority of terms (Fig 7B), while in the molecular function category, the top-represented GO terms included binding (GO:0005488) and catalytic activity (GO:0003824) (Fig 7C). The GO classifications suggested for *C*. *plicata* showed similarities with those for sequenced *P*. *yessoensis* [21] and *C*. *hongkongensis* [48].

**Fig 8:**
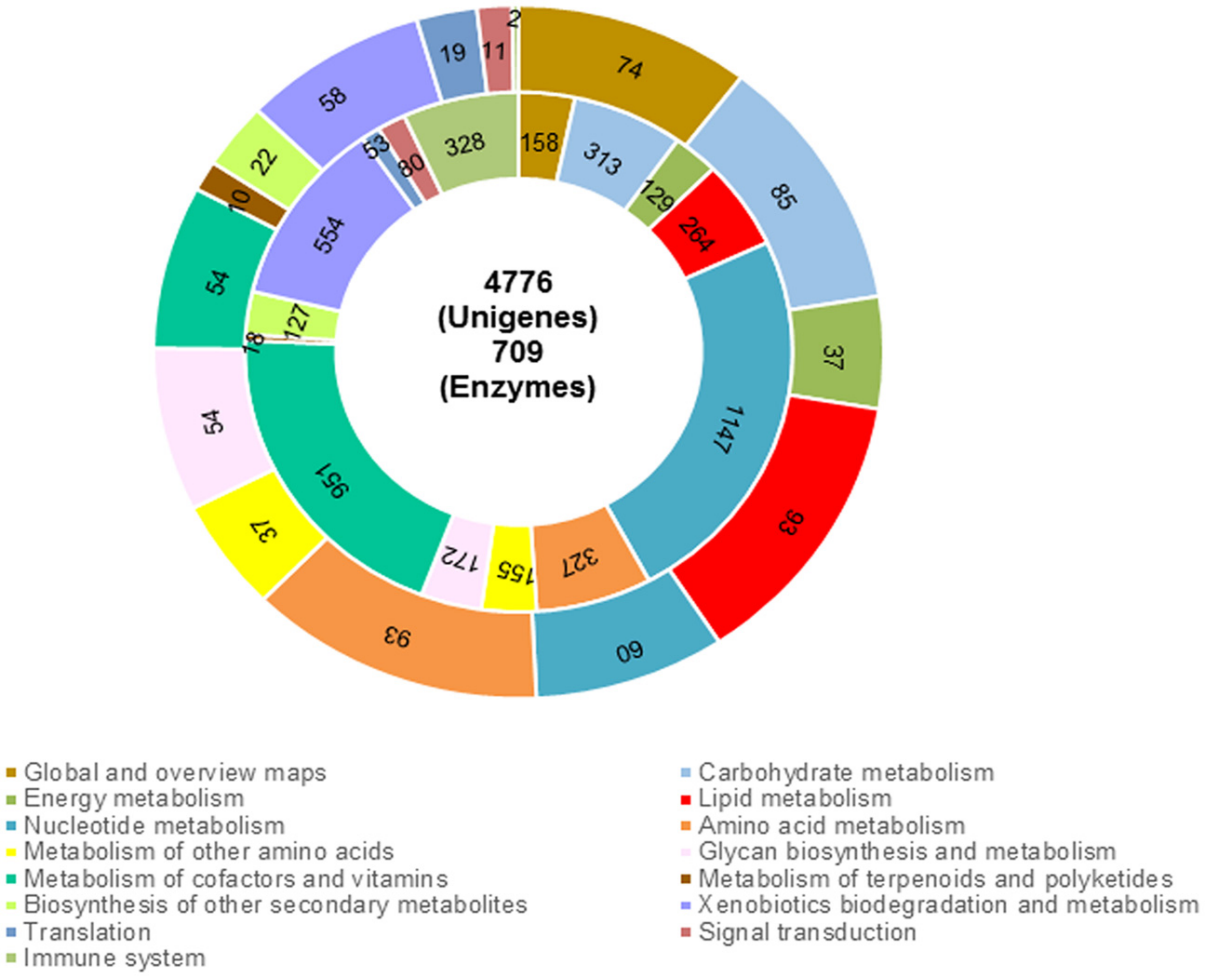
Kyoto Encyclopedia of Genes and Genomes (KEGG) pathway analysis. The *C*. *plicata* visceral mass unigenes were assigned to KEGG pathways (inner circle). The total number of enzymes ascribed within each KEGG pathway is shown in the outer circle. Each pathway is represented by a different color.

We also performed a search of all unigenes against the KEGG database to identify the active biological pathways in *C*. *plicata* Using BLASTx, we found 4,776 unigenes that shared homology with known enzymes in the KEGG database (Fig 8). The unigene sequences mapped to 123 KEGG pathways. Among them, 709 unigenes possessing an Enzyme Commission number were assigned to these pathways. The KEGG pathways were related to metabolism (4,315 unigenes), genetic information processing (53 unigenes), environmental information processing (80 unigenes), and organismal systems (328 unigenes). Predominantly, the unigenes were enriched in “nucleotide metabolism pathway” (1,147 unigenes of the 4,776 unigenes) followed by “metabolism of co-factors and vitamins” (951 unigenes), “xenobiotic biodegradation and metabolism” (554 unigenes), and “immune system pathways” (328 unigenes) categories. The *C*. *plicata* unigenes annotated to KEGG pathways are presented in S2 Table. Overall, both GO term and KEGG analyses identified, based on similarity to sequences already identified, transcribed region potentially involved in *C*. *plicata* stress responses as well as immunity and reproduction that could be crucial for determining adaptation in the natural environment and promoting conservation by means of genetic improvement programs.

### Genes and pathways related to the innate immune system

A combined annotation profile of the various methods allowed for an analysis of the *C*. *plicata* unigene sequences involved in immunity and stress mechanisms. We identified unigene sequences associated functionally with innate immunity and oxidative stress mechanisms. A summary of unigene sequences list of immunity and defense mechanisms is presented in S3 Table. A majority of unigenes showed homology to lectins, toll-like receptors (TLRs), and cathepsin, followed by complement C1q, scavenger receptor, caspase, and heat shock proteins (HSP). Overall, the *C*. *plicata* unigene sequences covered the major pathways and provided an extensive coverage of the immune gene repertoire in the species.

The innate immune system of molluscs consists of cellular and humoral components that operate in a coordinated manner to defend against a multitude of pathogens [64]. Pattern recognition proteins (PRPs) recognize microbial surfaces and initiate a signaling cascade which culminates in the regulatory release of antimicrobial effectors and forms the humoral defense response. The role of lectins as PRPs and in phagocytosis mechanisms has been characterized in molluscs [65,66] and is attributable mainly to the presence of carbohydrate-binding domains (CRDs) [67, 68]. The *C*. *plicata* transcriptome data shows the presence of putative lectin sequences including tandem-repeat galectin, C-type lectin, sialic-acid binding lectin, fucolectin, and immulectin-3. Tandem-repeat galectins are known to act as an acute-phase protein implicated in the immune defense of *R*. *philippinarum*, pearl oyster, and *Pinctada fucata* against *Vibrio* species [69,70]. C-type lectins have numerous roles in bivalve organisms such as non-self recognition, microbe agglutination, induction of phagocytosis and encapsulation, and anti-bacterial properties [71]. Transcriptome analysis of the related species, *C*. *virginica*, identified 8 galectins and 140 C-type lectin domain proteins [25]. A cross-species comparison analysis of C-type lectin domain proteins and CRD proteins showed an overrepresentation of such innate immune factors [25]. A comprehensive repertoire of carbohydrate-binding molecules has been analyzed in the common periwinkle, *Littorina littorea*, with unigene sequences corresponding to C-type lectins, fucolectins, galectins, chitinase-like lectins, and I-type lectins [72]. Fucolectins formed the largest group of CRD proteins in the *C*. *plicata* transcriptome. Fucolectins have been characterized in the mussel *Mytilus galloprovincialis* [73] and the sea cucumber *Apostichopus japonicus* [74], and have further characterized in detail [75]. Peptidoglycan recognition proteins (PGRPs), apolipophorin, and CD63, which are known for their recognition-mediated immune functions, were also identified among the C. plicata unigene sequences and may confer a selective advantage to the species under threatening conditions. Among the PGRPs, we identified both the short and long forms that bind to and hydrolyze bacterial peptidoglycans and activate the Toll or IMD signal transduction pathways in invertebrates [76,77]. PGRP homologs have been identified in squid (*Euprymna scolopes)* cDNA library [78] and the assembled transcriptomes of *C*. *virginica* [25] and *Octopus vulgaris* [50]. Apolipophorin III has been implicated in pattern recognition of beta-1, 3 glucans and responds to intracellular pathogens in insects [79,80], but requires a more extensive understanding in molluscs.

TLRs are membrane-bound pathogen recognition receptors implicated in intracellular signaling and they regulate the production of the effector antimicrobial peptides [81]. TLRs contain leucine-rich repeat motifs, a transmembrane region, and a cytoplasmic Toll/interleukin-1 receptor (TIR) domain which interacts with myeloid differentiation factor 88 (MyD88) or other adaptors, leading to activation of intracellular Toll signaling cascades. We identified TLR-2, -3, -4, -6, -7, -13, and other TLR precursors in the *C*. *plicata* transcriptome. In *M*. *edulis*, 27 TLRs have been described, indicative of diversity and an advanced immune system [19]. With a comprehensive array of TLRs identified from the transcriptome, it would be interesting to explore the innate immunity functions using a targeted gene approach. In addition, genes encoding intracellular Toll pathway proteins including MyD88, IRAK1 protein, relish, Janus kinase (JNK), and p38 have been identified from the rich transcriptome datasets, suggesting that the TLR pathway is conserved in *C*. *plicata*. Our results are consistent with TLR pathway genes identified in *M*. *galloprovincialis* (Illumina reads) [82], *M*. *edulis* (454 contigs and new assemblies) [83], and *C*. *gigas* (Illumina reads) [84]. Based on the TLR pathway in the related species *C*. *virginica*, the association of the TIR domain of TLR and MyD88 releases signals through IRAK and TRAF6 to induce the p38 signaling pathway (mediated by MEKK1, MKKs, or JNK) or the NF-κB-like relish protein. The identification of these intracellular components will increase our understanding of the TLR mediated immune system in *C*. *plicata*. MyD88 is an adaptor in the TLR/IL-1R signaling pathway and is highly expressed in bivalves in response to both Gram-positive and Gram-negative challenge. Recently, five genes encoding MyD88, which acts as an acute phase protein after infection with microbes, were characterized from *P*. *yessoensis* [85]. In an oyster model, TLR-based intracellular signaling is well represented, with mediators including MyD88, TNF (tissue necrosis factor) receptor associated factor 6 (TRAF6), and nuclear factor-κB (NF-κB) factors involved in the regulation of antimicrobial function [86]. Many gene families encoding proteins involved in immune responses to biotic and abiotic challenges have been identified from the C. gigas genome project including TLRs, MyD88, C-type lectins, fibrinogen-related proteins (FREP), superoxide dismutase (SOD), and globular head C1q domain containing protein (C1q) [87]. Transcriptome analysis of *C*. *virginica* [25] also revealed a rich set of genes related to the TLR pathway including *MyD88*, *SARM*, *IRAK*, *TRAF6*, *MKKs*, *JNK*, *p38*, *AP-1*, and *NF-κB*. We identified immune signaling candidates and immune effectors such as big defensins and defensins in the *C*. *plicata* transcriptome. Defensins are effectors of innate immunity present in marine bivalves [88] and some freshwater bivalves such as *Lamellidens marginalis* [89], *Hyriopsis schlegelii* [90], and *Hyriopsis cumingii* [91]. The evolution of antimicrobial peptide genes including defensins has been rapid in *Crassostrea* species, suggestive of molecular diversification of the effectors to cope with environmental challenges [92].

Cathepsins identified in this study are involved in the maintenance of homeostasis, regulation of antigen presentation and degradation, immune responses, and intracellular protein degradation. Eight putative cathepsin sequences (cathepsin B, C, D, F, I, L, S, and Z) present in the transcriptome may be required for several of highly regulated life processes. Multiple homologs and cDNA sequences of cathepsin L have been identified from *C*. *gigas* and *P*. *fucata* as well as a cloned cDNA from *C*. *plicata* [9,93,94]. The identification of candidate genes for environmental adaptation indicates that the dynamics of species survival should be further explored. Some unigene sequences in the *C*. *plicata* transcriptome showed homology to oxidative stress enzymes such as SOD (Mn-SOD and Cu-Zn SOD), glutathione-S-transferase (GST alpha, -mu, -omega, -pi, -sigma, -theta), catalase (CAT), glutathione peroxidase (GPX), and glutathione synthetase. The transcriptional stability of these genes is crucial under anthropogenic and pathogenic stresses and regulates cell homeostasis, as demonstrated in the mussel *M*. *galloprovincialis* [95]. SOD, CAT, and GPX were also identified in the *C*. *virginica* [25] and *L*. *fortunei* transcriptomes [17]. The heat shock protein (HSP) genes are key indicators of the physiological robustness of an organism and provide molecular insights into the response to environmental challenges [96]. The identification of HSP70 multigene family members and small HSP and HSP90 class genes reveals bivalve specialization for environmental sustainability. In the *C*. *plicata* transcriptome, we identified unigenes related putatively to HSP60, HSP70, and HSP90, and the small HSPs (HSP10, HSP20, and HSP40). HSP70 is associated with acclimation robustness in molluscs and is found to be highly expressed in response to biotic stressors [17,97]. Differential, tissue- and time-specific expression of HSP70 in *C*. *hongkongensis* [98] and HSP60 in the mussel *Perna viridis* [99] has been reported. HSP chaperones are important factors for the establishment of mussels such as *M*. *galloprovincialis* and *M*. *trossulus* in North America, and are proposed to serve as a biochemical marker [100].

Analysis of the *C*. *plicata* transcriptome also revealed several key genes encoding proteins related to apoptosis and programmed cell death including caspases (caspase-1, -2, -3, -7, -8, -9 like isoform, -10), Bcl-2, and Bax, suggestive of the significance of apoptosis in the immune response of the organism. The abundance of unigenes showing homology to caspases was apparent from the identification of initiator caspase group 2 and 8 and executioner caspase group 3 and 7. The appearance of multiple executioner caspases and the divergence in their sequences compared to the conserved initiator caspases reflects the importance of the executioner phase of apoptosis in bivalves [101]. A similar set of key apoptosis genes was identified in the related species, *C*. *virginica*, suggestive of a versatile apoptosis-pathway mediating system [25]. In accordance with an earlier report of the presence of C1q domain proteins in the *C*. *virginica*, *M*. *galloprovincialis*, and *M*. *edulis* transcriptomes, we identified a large number of C1q domain proteins in the *C*. *plicata* transcriptome, which supports the expansion of such proteins in bivalve molluscs, possibly in support of adaptation strategies [19,25,102].

### Sex-determination and reproduction-related genes

Genes involved in the sex determination process determine the sex of an organism by directing the development of gonadal structures, such as the ovary or testis. Sex-differentiation genes regulate the development of the ovaries and the testis from the undifferentiated gonad. Gonad transcriptome analysis studies have taken advantage of the diverse reproduction strategies of molluscs to identify a large number of sex determination/differentiation genes [48,103–106]. The economic advantages of *C*. *plicata* for aquaculture industries and its endangered status make exploring the molecular mechanisms underlying gametogenesis important. We identified unigene sequences showing homology to sex determination, sex differentiation, and reproduction-related genes based on a keyword search of the PANM-DB using our BLASTx annotation results (S4 Table). The *C*. *plicata* unigenes showed homology to sex-determination genes including SRY-related HMG-box domain (*SOX*) family members (*SOX5*, *SOX6*, *SOX9*, *SOX11*, *SOX15*, and *SOXB2*), Doublesex, and mab-3 related transcription factors (*DMRT3*), WNT member 4 (*WNT4*), Fem-1 like protein (*FEM1*), steroidogenic factor 1 (*SF1*), sex-determining region on the Y-chromosome (*SRY*), dosage-sensitive sex reversal, adrenal hypoplasia critical region, and on chromosome X (gene 1, DAX1). Sex determination and differentiation genes that were conspicuously absent from the *C*. *plicata* transcriptome included Wilm’s tumor suppressor gene-1 (*WT-1*), forkhead box L2 (*FOXL2*), aromatase (CYP19A1), and anti-mullerian hormone. While WT-1 homologs have been reported in fish (*Danio rerio)*, sturgeons (*A*. *sinensis and A*. *naccarii*), amphibians (*H*. *chinensis*), and mouse (*Mus musculus*), they have not been reported in oyster (*C*. *hongkongensis)* [48]. Homologs of aromatase and anti-mullerian hormone from oyster species have also not been reported. *FOXL2* is an ovarian determination gene in vertebrates and it functions to suppress genes involved in testis differentiation. Homologs of *FOXL2* show higher expression in ovaries of invertebrates, including molluscs [25,105,107]. Homologs may be present in *C*. *plicata*, suggestive of a role in the earlier stages of gonad development. In *C*. *hongkongensis*, sex determination genes such as *DMRT1* and *SRY* were absent while homologs of other *DMRT* and *SOX* family members were present [48]. Genes including *DMRT*, *SOX9*, *Fem1*, and *FOXL2* are known to participate in the regulatory processes underlying sex determination/differentiation [108,109]. SOX family proteins are a conserved group of transcription regulators involved in development and differentiation which possess a high mobility group (HMG)-box domain [29,110]. *SRY* (founding member of the SOX genes and the master switch in sex determination) with *SOX9* activates the male determining pathway by inhibiting ovary development through the induction of anti-mullerian hormone in Sertoli cells [111,112]. A *DMRT-1* testis-specific (zinc finger DM domain protein) unigene sequence was identified among the *C*. *plicata* unigenes, and may (along with other members of the family) promote male-specific development. This finding is supported by reports of testis-specific DMRT genes in other molluscs [105,113,114]. Other genes such as *FEM1* and *WNT4* are not involved in the sex-determining pathway or maintenance of mature gonads but may have a role at an earlier stage of sex-specific expression [25].

For the conservation of endangered species such as *C*. *plicata*, it is important to understand reproduction-specific unigenes from the transcriptome. We identified unigene sequences homologous to spermatogenesis-associated protein (SPATA1, 4, 5, 6 and 7), sperm flagellar protein 1/2, sperm motility kinase, nuclear autoantigenic sperm protein, spermidine synthase, and spermine oxidase potentially expressed in the male reproductive tissues. As expected, the majority of male reproduction-related genes are associated with sperm motility, which is crucial for the reproductive success of an organism. Moreover, we identified *C*. *plicata* unigenes showing homology to oocyte zinc finger protein, ovary development protein, and vitellogenin (Vg) which are other putative reproduction-related genes. Vg has a strong transcript presence in the ovary compared to the testis and is an important protein for oocyte maturation in molluscan species such as *C*. *gigas* [115], *P*. *yessoensis* [116], and *Argopecten purpuratus* [117]. Overall, the *C*. *plicata* transcriptome was useful for the discovery of homologs related to sex-determination and reproduction from among the assembled unigene sequences.

### Characterization of cSSR markers in the *C*. *plicata* transcriptome

**Fig 9:**
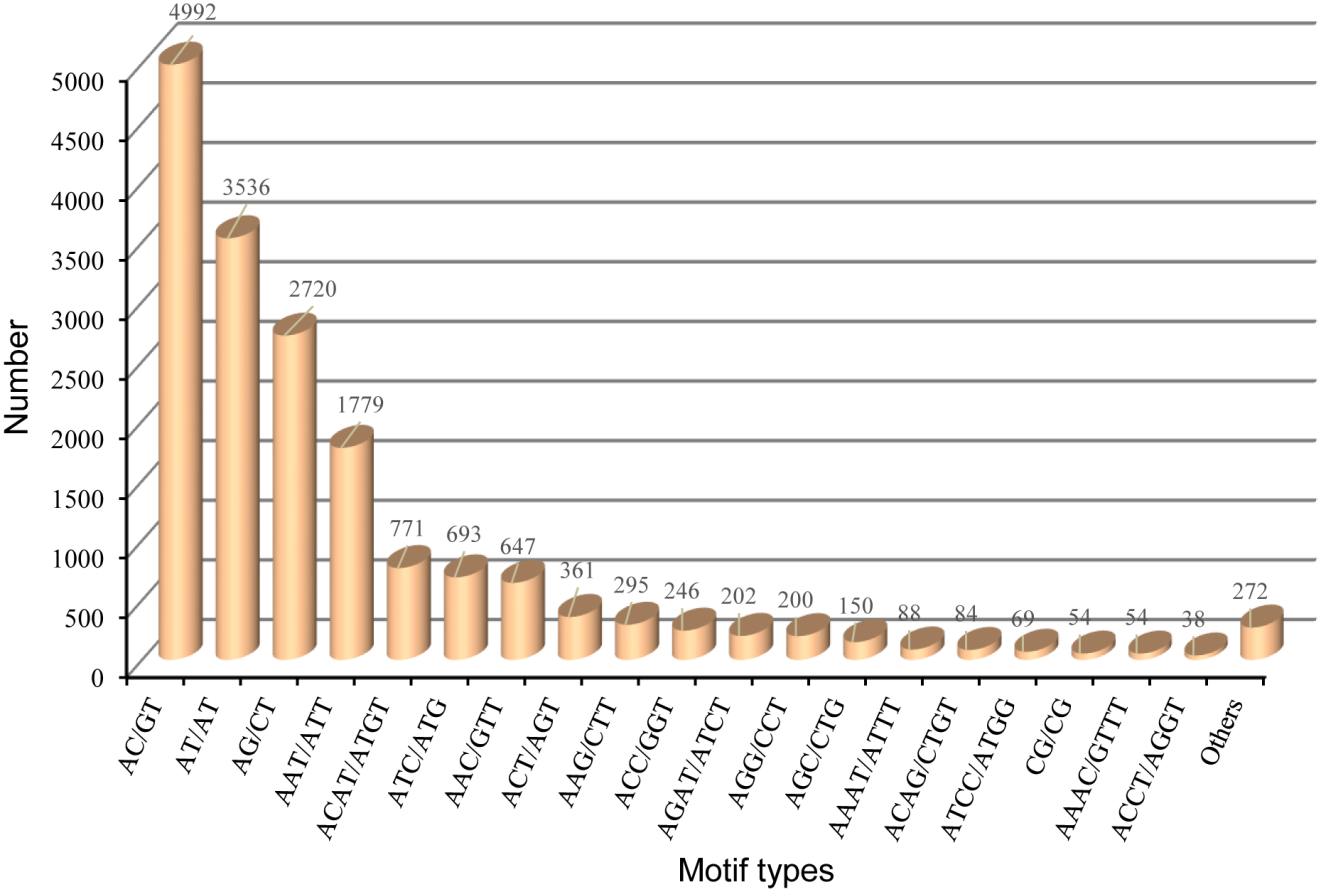
Frequency distribution of simple sequence repeats (SSRs) based on motif types found in *C*. *plicata* visceral mass unigene sequences.

SSRs are tandem repeated motifs characterized by high-levels of polymorphism and are commonly used as marker systems in genetic diversity assessments, population structure dynamics studies, conservation genomics, and genetic linkage mapping for a variety of organisms [118–121]. These microsatellite sequences can be important for the characterization of invasive species with reduced genetic diversity [122–123] and can resolve structural dynamics of closely related populations [124]. Unfortunately, due to limitations of time and cost of development, microsatellite isolation from non-model organisms remains limited. Recently, with the availability of genomic and transcriptome sequences using the high-throughput and cost-efficient NGS platforms, *de novo* screening of large sets of microsatellites such as SSRs and single nucleotide polymorphism (SNPs) has become more common in non-model organisms [125–126], including mollusc species [48, 127]. Due to the extensive use of *C*. *plicata* as a pearl culture resource in China, few polymorphic microsatellite loci were developed using the dinucleotide-enriched genomic library for the improvement of species [128]. To address the conservation efforts of *C*. *plicata*, which is endangered in Korea, the development of SSRs is highly desirable. We obtained a set of SSRs from the transcriptomic dataset using the MISA program. A total of 61,141 unigene sequences (>1 kb) containing 17,251 SSRs were identified, with 3269 sequences containing more than one cSSR. We screened for dinucleotide repeats with a minimum of six iterations, and all other repeat types (tri- to hexanucleotides) repeated at least five times. The most abundant SSRs included dinucleotide repeats (11,302), followed by tri- (4381), tetra- (1527), penta- (40), and hexanucleotide repeats (1). Consistent with our results, dinucleotide repeats were the most abundant repeats in the cSSR profiles of *M*. *rosenbergii* [16], endangered *A*. *sinensis* [30], and Chinese salamander (*H*. *chinensis*) [29]. Conversely, in the invasive *L*. *fortunei*, tetranucleotide repeats were more common while dinucleotide repeats were less common [17]. A summary of SSRs based on the number of repeat is presented in Table 5. Six tandem repeats (3635) were predominant, followed by five (2479) and seven (2111) tandem repeats. The cSSRs also contained repeats with 20 (216) and ≥ 21 (1595) random reiterations consisting mostly of dinucleotide repeats (89.66%). An analysis of the frequency distribution of cSSRs based on motif sequence types is shown in Fig 9. The *C*. *plicata* transcriptome is rich in AC/GT (4992; 28.94%), AT/AT (3536; 20.50%) and AG/CT (2720; 15.77) repeats. The abundant repeats in the cDNA-associated SSRs of *C*. *hongkongensis* were identified as AG followed by AT, AC, and ATC [48]. The prominent trinucleotide repeat types common among cSSRs of *C*. *plicata* included AAT/ATT (1779; 10.31%), followed by ATC/ATG (693; 4.02%), AAC/GTT (647; 3.75%), ACT/AGT (361; 2.09%), and AAG/CTT (295; 1.71%). A tetranucleotide repeat ACAT/ATGT (771; 4.47%) was also identified within the top prominent SSR types. Generally, microsatellite repeat types exhibit species-specific differences, as has been reported for Crustacean species [16,129]. SSRs identified in this study can be valuable for genetic improvement programs and for the quantification of genetic diversity within and among populations of endangered *C*. *plicata*.

**Table 5:** Summary of simple sequence repeat (SSR) types based on the number of repeat units.

Repeat numbers	Motif length					Total
	di-	tri-	tetra-	penta-	hexa-	
5	0	1936	526	17	0	2479
6	2541	713	380	1	0	3635
7	1648	417	44	2	0	2111
8	1211	401	66	1	0	1679
9	751	104	60	2	0	917
10	652	117	50	4	0	823
11	853	102	29	2	0	986
12	618	79	37	1	0	735
13	189	57	44	2	0	292
14	263	86	32	3	0	384
15	237	46	44	1	1	329
16	220	48	45	0	0	313
17	201	52	39	0	0	292
18	204	38	32	2	0	276
19	127	43	19	0	0	189
20	157	46	13	0	0	216
≥21	1430	96	67	2	0	1595
Total	11302	4381	1527	40	1	17251

## Conclusions

This study is the first exhaustive investigation of the transcriptome of the endangered freshwater pearl bivalve, *C*. *plicata*. Using the Illumina HiSeq 2500 NGS platform and the Trinity assembler, we assembled approximately 79,960 unigenes and assigned them to 23,246 GO and 4,776 KEGG pathway annotations. A number of candidate unigenes involved in immunity, sex determination/differentiation, and reproduction were identified. Transcriptome sequence and annotation data are valuable because *C*. *plicata* has been listed as endangered in the Korean Red List of Threatened Species due to a decline in their natural habitat over time. Genetic markers in the form of cSSRs may assist in the development of genetic improvement programs for *C*. *plicata*.

## Supporting Information

S1 Table Mapping of the annotated unigenes against *C*. *plicata* mitochondrial protein genes using BLASTn at a cutoff E-value of 1E-5. (DOCX)

S2 Table Annotation of *C*. *plicata* unigenes to KEGG pathways. (XLSX)

S3 Table Genes of interest for immune signaling response and defense mechanisms in *C*. *plicata* transcriptome. (DOCX)

S4 Table Candidate genes for sex-determination and reproduction in *C*. *plicata* transcriptome. (DOCX)

## References

[1] Dong ZG, Li L.J. Biodiversity and conservation of freshwater mollusks. Acta Hydrobiologica Sinica. 2004; 4: 440–444.

[2] Kondo T. Monograph of Unionoida in Japan (Mollusca: Bivalvia). Special publication of the Malacological Society of Japan 3: v–69.

[3] He J, Zhuang Z. The freshwater bivalves of China. ConchBooks, Harxheim, Germany.

[4] Bogan AE, Cummings K. Cristaria plicata. The IUCN Red List of Threatened Species. Version 2015.2. www.iucnredlist.org.

[5] Lee JH, Choi EH, Kim SK, Ryu SH, Hwang UW. Mitochondrial genome of the cockscomb pearl mussel Cristaria plicata (Bivalvia, Unionoida, Unionidae). Mitochond DNA. 2012; 23: 39–41. 10.3109/19401736.2011.643882 22295867

[6] Wang H, He L, Yang X, Yang S, Li C, Wang X. Determination of the complete mitochondrial genome sequence of mussel Cristaria plicata (Leach). Mitochond DNA. 2014 10.3109/19401736.2014.953100 25162301

[7] Yang HJ, Li G, Wen CG, Hu BQ, Deng LR, Pei PZ, et al A catalase from the freshwater mussel Cristaria plicata with cloning, identification and protein characterization. Fish Shellfish Immun. 2011; 31: 389–399. 10.1016/j.fsi.2011.06.003 21689759

[8] Wu D, Hu B, Wen C, Lin G, Tao Z, Hu X, et al Gene identification and recombinant protein of a lysozyme from freshwater mussel *Cristaria plicata*. Fish Shellfish Immun. 2013; 34: 1033–1041. 10.1016/j.fsi.2012.12.009 23333359

[9] Hu X, Hu X, Hu B, Wen C, Xie Y, Wu D, et al Molecular cloning and characterization of cathepsin L from freshwater mussel, Cristaria plicata. Fish Shellfish Immun. 2014; 40: 446–454. 10.1016/j.fsi.2014.07.005 25038281

[10] Birol I, Behsaz B, Hammond SA, Kucuk E, Veldhoen N, Helbing CC. De novo transcriptome assemblies of Rana (Lithobates) catesbeiana and Xenopus laevis tadpole livers for comparative genomics without reference genomes. PLoS ONE. 2015; 10(6): e0130720 10.1371/journal.pone.0130720 26121473 PMC4488148

[11] Mehr S, Verdes A, DeSalle R, Sparks J, Pieribone V, Gruber DF. Transcriptome sequencing and annotation of the polychaete Hermodice carunculata (Annelida, Amphinomidae). BMC Genomics. 2015; 16: 445 10.1186/s12864-015-1565-6 26059236 PMC4462082

[12] Hook SE, Osborn HL, Spadaro DA, Simpson SL. Assessing mechanism of toxicant response in the amphipod *Melita plumulosa* through transcriptomic profiling. Aquat Toxicol. 2014; 146: 247–257. 24334007 10.1016/j.aquatox.2013.11.001

[13] Qiao L, Yang W, Fu J, Song Z. Transcriptome profile of the Green Odorous Frog (Odorrana margaretae). PLoS ONE. 2013; 8(9): E75211 10.1371/journal.pone.0075211 24073255 PMC3779193

[14] Micallef G, Bickerdike R, Reiff C, Fernandes JM, Bowman AS, Martin SA. Exploring the transcriptome of Atlantic salmon (Salmo salar) skin, a major defense organ. Mar Biotechnol. 2012; 14: 559–569. 10.1007/s10126-012-9447-2 22527268

[15] Zeng D, Chen X, Xie D, Zhao Y, Yang C, Li Y, et al Transcriptome analysis of Pacific White Shrimp (Litopenaeus vannamei) hepatopancreas in response to Taura Syndrome Virus (TSV) experimental infection. PLoS ONE. 2013; 8(2): e57515 10.1371/journal.pone.0057515 23469011 PMC3585375

[16] Jung H, Lyons RE, Dinh H, Hurwood DA, McWilliam S, Mather PB. Transcriptomics of a Giant Freshwater Prawn (Macrobrachium rosenbergii): De novo assembly, annotation and marker discovery. PLoS ONE. 2011; 6(12): e27938 10.1371/journal.pone.0027938 22174756 PMC3234237

[17] Uliano-Silva M, Americo JA, Brindeiro R, Dondero F, Prosdocimi F, de Freitas Rebelo M. Gene discovery through transcriptome sequencing for the invasive mussel *Limnoperna fortunei*. PLoS ONE. 2014; 9(7): e102973 10.1371/journal.pone.0102973 25047650 PMC4105566

[18] Huang Z-X, Chen Z-S, Ke C-H, Zhao J, You W-W, Zhang J, et al Pyrosequencing of *Haliotis diversicolor* transcriptomes: Insights into early developmental molluscan gene expression. PLoS ONE. 2012; 7(12): e51279 10.1371/journal.pone.0051279 23236463 PMC3517415

[19] Philipp EER, Kraemer L, Melzner F, Poustka AJ, Thieme S, Findeisen U, et al Massively parallel RNA sequencing identifies a complex immune gene repertoire in the lophotrochozoan *Mytilus edulis*. PLoS ONE. 2012; 7(3): e33091 10.1371/journal.pone.0033091 22448234 PMC3308963

[20] Milan M, Coppe A, Reinhardt R, Cancela LM, Leite RB, Saavedra C, et al Transcriptome sequencing and microarray development for the Manila clam, *Ruditapes philippinarum*: genomic tools for environmental monitoring. BMC Genomics. 2011; 12: 234 10.1186/1471-2164-12-234 21569398 PMC3107815

[21] Hou R, Bao Z, Wang S, Su H, Li Y, Du H, et al Transcriptome sequencing and De Novo analysis for Yesso Scallop (Patinopecten yessoensis) using 454 GS FLX. PLoS ONE. 2011; 6(6): e21560 10.1371/journal.pone.0021560 21720557 PMC3123371

[22] Clark MS, Thorne MAS, Vieira FA, Cardoso JCR, Power DM, Peck LS. Insights into shell deposition in the Antartic bivalve *Laternula elliptica*: gene discovery in the mantle transcriptome using 454 pyrosequencing. BMC Genomics. 2010; 11: 362 10.1186/1471-2164-11-362 20529341 PMC2896379

[23] Prentis PJ, Pavasovic A. The Anadara trapezia transcriptome: a resource for molluscan physiological genomics. Mar Genomics. 2014; Pt B: 113–115. 10.1016/j.margen.2014.08.004 25151889

[24] Meng X-L, Liu M, Jiang K-Y, Wang B-J, Tian X, Sun S-J, et al *De Novo* characterization of Japanese scallop *Mizuhopecten yessoensis* transcriptome and analysis of its gene expression following cadmium exposure. PLoS ONE. 2013; 8(5): e64485 10.1371/journal.pone.0064485 23741332 PMC3669299

[25] Zhang L, Li L, Zhu Y, Zhang G, Guo X. Transcriptome analysis reveals a rich gene set related to innate immunity in the Eastern Oyster (Crassostrea virginica). Mar Biotechnol. 2014; 16: 17–33. 10.1007/s10126-013-9526-z 23907648

[26] Franchini P, van der Merwe M, Roodt-Wilding R. Transcriptome characterization of the South African abalone Haliotis midae using sequencing-by-synthesis. BMC Res Notes. 2011; 4: 59 10.1186/1756-0500-4-59 21396099 PMC3063225

[27] Feldmeyer B, Wheat CW, Krezdorn N, Rotter B, Pfenninger M. Short read Illumina data for the de novo assembly of a non-model snail species transcriptome (Radix balthica, Basommatophora, Pulmonata), and a comparison of assembler performance. BMC Genomics. 2011; 12: 317 10.1186/1471-2164-12-317 21679424 PMC3128070

[28] Smith S, Wilson NG, Goetz F, Feehery C, Andrade SCS, Rouse GW, et al Resolving the evolutionary relationships of molluscs with phylogenomics tools. Nature. 2011; 480: 364–367. 10.1038/nature10526 22031330

[29] Che R, Sun Y, Wang R, Xu T. Transcriptomic analysis of endangered Chinese Salamander: Identification of Immune, Sex and Reproduction-related genes and Genetic Markers. PLoS ONE, 2014; 9(1): E87940 10.1371/journal.pone.0087940 24498226 PMC3909259

[30] Yue H, Li C, Du H, Zhang S, Wei Q. Sequencing and De Novo assembly of the Gonadal transcriptome of the endangered Chinese Sturgeon (Acipenser sinensis). PLoS ONE. 2015; 10(6): e0127332 10.1371/journal.pone.0127332 26030930 PMC4452307

[31] Joshi NA, Fass JN. Sickle: A sliding-window, adaptive, quality-based trimming tool for FastQ files (Version 1.33) [Software]. Available at https://github.com/najoshi/sickle.

[32] Blankenberg D, Gordon A, Von Kuster G, Coraor N, Taylor J, Nekrutenko A, et al Manipulation of FASTQ data with Galaxy. Bioinformatics. 2010; 26(14): 1783–1785. 10.1093/bioinformatics/btq281 20562416 PMC2894519

[33] Haas BJ, Papanicolaou A, Yassour M, Grabherr M, Blood PD, Bowden J, et al De Novo transcript reconstruction from RNA-seq using the Trinity platform for reference generation and analysis. Nat Prot. 2013; 8: 1494–1512. 10.1038/nprot.2013.084 PMC3875132 23845962

[34] Pertea G, Huang X, Liang F, Antonescu V, Sultana R, Karamycheva S, et al TIGR Gene Indices Clustering Tool (TGICL): a software system for fast clustering of large EST datasets. Bioinformatics. 2003; 19: 651–652. 10.1093/bioinformatics/btg034 12651724

[35] Kang SW, Park SY, Patnaik BB, Hwang HJ, Kim C, Kim S, et al Construction of PANM database (Protostome DB) for rapid annotation of NGS data in mollusks. Korean J Malacol. 2015; 31(3): 243–247. 10.9710/kjm.2015.31.3.243

[36] Altschul SF, Gish W, Miller W, Myers EW, Lipman DJ. Basic local alignment search tool. J Mol Biol. 1990; 215(3): 403–410. 2231712 10.1016/S0022-2836(05)80360-2

[37] Consea A, Gotz S, Garcia-Gomez JM, Terol J, Talon M, Robles M. Blast2go: a universal tool for annotation, visualization and analysis in functional genomics research. Bioinformatics. 2005; 21: 3674–3676. 16081474 10.1093/bioinformatics/bti610

[38] The.gene.ontology.consortium. The gene ontology project in 2008. Nucleic Acids Res. 2008; 36 (Database issue). 10.1093/nar/gkm883 PMC2238979 17984083

[39] Kanehisa M, Goto S, Kawashima S, Okuno Y, Hattori M. The KEGG resource for deciphering the genome. Nucleic Acids Res. 2004; 32(Database issue): D277–280. 14681412 10.1093/nar/gkh063 PMC308797

[40] Zdobnov EM, Apweiler R. InterProScan-an integration platform for the signature-recognition methods in InterPro. Bioinformatics. 2001; 17(9): 847–848. 11590104 10.1093/bioinformatics/17.9.847

[41] Tatusov RL, Galperin MY, Natale DA, Koonin EV. The COG database: a tool for genome-scale analysis of protein functions and evolution. Nucleic Acids Res. 2000; 28: 33–36. 10592175 10.1093/nar/28.1.33 PMC102395

[42] Benson G. Tandem Repeats finder: a program to analyze DNA sequences. Nucleic Acids Res. 1999; 27: 573–580. 9862982 10.1093/nar/27.2.573 PMC148217

[43] Grabherr MG, Haas BJ, Yassour M, Levin JZ, Thompson DA, Amit I, et al Full-length transcriptome assembly from RNA-Seq data without a reference genome. Nat Biotechnol. 2011; 29: 644–652. 10.1038/nbt.1883 21572440 PMC3571712

[44] Schulz MH, Zerbino DR, Vingron M, Birney E. Oases: robust *de novo* RNA-seq assembly across the dynamic range of expression levels. Bioinformatics. 2012; 28(8): 1086–1092. 10.1093/bioinformatics/bts094 22368243 PMC3324515

[45] Robertson G, Schein J, Chiu R, Corbett R, Field M, Jackman SD et al De novo assembly and analysis of RNA-seq data. Nat Methods. 2010; 7: 909–912. 10.1038/nmeth.1517 20935650

[46] Xie Y, Wu G, Tang J, Luo R, Patterson J, Liu S et al SOAPdenovo-Trans: *De novo* transcriptome assembly with short RNA-Seq reads. Bioinformatics. 2014; 30(12): 1660–1666. 10.1093/bioinformatics/btu077 24532719

[47] Martin J, Bruno VM, Fang Z, Meng X, Blow M, Zhang T et al Rnnotator: an automated *de novo* transcriptome assembly pipeline from stranded RNA-Seq reads. BMC Genomics. 2010; 11:663 10.1186/1471-2164-11-663 21106091 PMC3152782

[48] Tong Y, Zhang Y, Huang J, Xiao S, Zhang Y, Li J et al Transcriptomics analysis of Crassostrea hongkongensis for the discovery of reproduction-related genes. PLoS ONE. 2015; 10:e0134280 10.1371/journal.pone.0134280 26258576 PMC4530894

[49] Senatore A, Edirisinghe N, Katz PS. Deep mRNA sequencing of the Tritonia diomedea brain transcriptome provides access to gene homologues for neuronal excitability, synaptic transmission and peptidergic signaling. PLoS ONE. 2015; 10(2):e0118321 10.1371/journal.pone.0118321 25719197 PMC4342343

[50] Castellanos-Martinez S, Artela D, Catarino S, Gestal C. De novo transcriptome sequencing of the Octopus vulgaris hemocytes using Illumina RNA-Seq technology: response to the infection by the gastrointestinal parasite Aggregata octopiana. PLoS ONE. 2014; 9(10):e107873 10.1371/journal.pone.0107873 25329466 PMC4199593

[51] Gerdol M, De Moro G, Manfrin C, Milandri A, Riccardi E, Beran A et al RNA sequencing and de novo assembly of the digestive gland transcriptome in Mytilus galloprovincialis fed with toxinogenic and non-toxic strains of *Alexandrium minutum*. BMC Res Notes. 2014; 7:722 10.1186/1756-0500-7-722 25314922 PMC4203926

[52] Leung PTY, Ip JCH, Mak SST, Qiu JW, Lam PKS, Wong CKC et al *De novo* transcriptome analysis of *Perna viridis* highlights tissue-specific patterns for environmental studies. BMC Genomics. 2014; 15:804 10.1186/1471-2164-15-804 25239240 PMC4190305

[53] Deng Y, Lei Q, Tian Q, Xie S, Du X, Li J et al De novo assembly, gene annotation, and simple sequence repeat marker development using Illumina paired-end transcriptome sequences in the pearl oyster *Pinctada maxima*. Biosci Biotechnol Biochem. 2014; 78(10): 1685–1692. 10.1080/09168451.2014.936351 25047366

[54] Wang W, Hui JHL, Chan TF, Chu KH. *De novo* transcriptome sequencing of the snail *Echinolittorina malaccana*: Identification of genes responsive to thermal stress and development of genetic markers for population studies. Mar Biotechnol. 2014; 16(5): 547–559. 10.1007/s10126-014-9573-0 24825364

[55] Artigaud S, Thorne MAS, Richard J, Lavaud R, Jean F, Flye-Sainte-Marie J. Deep sequencing of the mantle transcriptome of the great scallop *Pecten maximus*. Mar Genomics. 2014; 15:3–4. 10.1016/j.margen.2014.03.006 24731930

[56] Pauletto M, Milan M, Moreira R, Novoa B, Figueras A, Babbucci M et al Deep transcriptome sequencing of *Pecten maximus* hemocytes: A genomic resource for bivalve immunology. Fish Shellfish Immunol. 2014; 37:154–165. 10.1016/j.fsi.2014.01.017 24486903

[57] Chen H, Zha J, Liang X, Bu J, Wang M, Wang Z. Sequencing and De novo assembly of the Asian clam (Corbicula fluminea) transcriptome using the Illumina GAIIx method. PLoS ONE. 2013; 8(11):e79516 10.1371/journal.pone.0079516 24244519 PMC3820681

[58] Shi M, Lin Y, Xu G, Xie L, Hu X, Bao Z et al Characterization of the Zhikong scallop (Chlamys farreri) mantle transcriptome and identification of biomineralization-related genes. Mar Biotechnol. 2013; 15:706–715. 10.1007/s10126-013-9517-0 23860577

[59] Niu D, Wang L, Sun F, Liu Z, Li J. Development of molecular resources for an intertidal clam, Sinonovacula constricta using 454 transcriptome sequencing. PLoS ONE. 2013; 8(7):e674456 10.1371/journal.pone.0067456 PMC3723811 23935831

[60] Bai Z, Yuan Y, Yue G, Li J. Molecular cloning and copy number variation of a ferritin subunit (Fth1) and its association with growth in freshwater pearl mussel Hyriopsis cumingii. PLoS ONE. 2011; 6(7): e22886 10.1371/journal.pone.0022886 21818403 PMC3144951

[61] Pairett AN, Serb JM. De novo assembly and characterization of two transcriptomes reveal multiple light-mediated functions in the scallop eye (Bivalvia: Pectinidae). PLoS ONE. 2013; 8(7): e69852 10.1371/journal.pone.0069852 23922823 PMC3726758

[62] Bouhouche N, Syvanen M, Kado CI. The origin of the prokaryotic C2HC zinc finger regulators. Trends Microbiol. 2000; 8: 77–81. 10664601 10.1016/s0966-842x(99)01679-0

[63] Rhee SY, Wood V, Dolinski K, Draghici S. Use and misuse of the gene ontology annotations. Nat Rev Genet. 2008; 509–515. 10.1038/nrg2363 18475267

[64] Galloway TS, Depledge MH. Immunotoxicity in invertebrates: Measurement and ecotoxicological relevance. Ecotoxicology. 2001; 10; 5–23. 11227817 10.1023/a:1008939520263

[65] Wang L, Wang L, Huang M, Zhang H, Song L. The immune role of C-type lectins in molluscs. ISJ. 2011; 8: 241–246.

[66] Chatterjee BP, Adhya M. Lectins with varying specificity and biological activity from marine bivalves. Mar Proteins and Peptides: Biological activities and applications. 2013; 41–68.

[67] Kuchel RP, Aladaileh S, Birch D, Vella N, Raftos DA. Phagocytosis of the protozoan parasite, Marteilia sydneyi, by Sydney rock oyster (Saccostrea glomerata) hemocytes. J Invertebr Pathol. 2010; 104: 97–104. 20153334 10.1016/j.jip.2010.02.001

[68] Cheng C-F, Hung S-W, Chang Y-C, Chen M-H, Chang C-H, Tsou L-T et al Purification and characterization of hemagglutinating proteins from Poker-Chip Venus (Meretrix lusoria) and Corbicula Clam (Corbicula fluminea). The Scientific World J. 2012 10.1100/2012/906737 PMC3361307 22666167

[69] Kim JY, Kim YM, Cho SK, Choi KS, Cho M. Noble tandem-repeat galectin of Manila clam Ruditapes philippinarum is induced upon infection with the protozoan parasite *Perkinsus olseni*. Dev Comp Immunol. 2008; 32: 1131–1141. 10.1016/j.dci.2008.03.002 18440068

[70] Zhang DC, Hu YT, Guo HY, Cui SG, Su TF, Jiang SG. cDNA cloning and mRNA expression of a tandem-repeat galectin (PoGal2) from the pearl oyster, Pinctada fucata. Genet Mol Res. 2011; 10: 1963–1974. 21948759 10.4238/vol10-3gmr1149

[71] Kong P, Wang L, Zhang H, Song X, Zhou Z, Yang J et al A novel C-type lectin from bay scallop Argopecten irradians (AiCTL-7) agglutinating fungi with mannose specificity. Fish Shellfish Immunol. 2011; 30: 836–844. 21255651 10.1016/j.fsi.2011.01.005

[72] Gorbushin AM, Borisova EA. Lectin-like molecules in transcriptome of *Littorina littorea* hemocytes. Dev Comp Immunol. 2015; 48(1): 210–220. 10.1016/j.dci.2014.10.007 25451301

[73] Venier P, De Pitta C, Bernante F, Varotto L, De Nardi B, Bovo G et al MytiBase: a knowledge base of mussel (M. galloprovincialis) transcribed sequences. BMC Genomics. 2009; 10:72 10.1186/1471-2164-10-72 19203376 PMC2657158

[74] Dong Y, Sun H, Zhou Z, Yang A, Chen Z, Guan X et al Expression analysis of immune related genes identified from the coelomocytes of Sea Cucumber (Apostichopus japonicus) in response to LPS challenge. Int J Mol Sci. 2014; 15(11): 19472–19486. 10.3390/ijms151119472 25421239 PMC4264123

[75] Vasta GR, Ahmed H, Bianchet MA, Fernandez-Robledo JA, Amzel LM. Diversity in recognition of glycans by F-type lectins and galectins: molecular, structural, and biophysical aspects. Ann N Y Acad Sci. 2012; 1253: E14–E26. 10.1111/j.1749-6632.2012.06698.x 22973821 PMC3683447

[76] Vogel H, Altincicek B, Glockner G, Vilcinskas A. A comprehensive transcriptome and immune-gene repertoire of the lepidopteran model host *Galleria mellonella*. BMC Genomics. 2011; 12:308 10.1186/1471-2164-12-308 21663692 PMC3224240

[77] Tindwa H, Patnaik BB, Kim DH, Mun S, Jo YH, Lee BL et al Cloning, characterization and effect of TmPGRP-LE gene silencing on survival of *Tenebrio molitor* against Listeria monocytogenes infection. Int J Mol Sci. 2013; 14(11): 22462–22482. 10.3390/ijms141122462 24240808 PMC3856074

[78] Collins AJ, Schleicher TR, Rader BA, Nyholm SV. Understanding the role of host hemocytes in a squid/*Vibrio* symbiosis using transcriptomics and proteomics. Front Immunol. 2012; 3:91 10.3389/fimmu.2012.00091 22590467 PMC3349304

[79] Whitten MMA, Tew IF, Lee BL, Ratcliffe NA. A novel role for an insect apolipoprotein (apolipophorin III) in β-1,3-glucan pattern recognition and cellular encapsulation reactions. J Immunol. 2004; 2177–2185. 10.4049/jimmunol.172.4.2177 14764684

[80] Noh JY, Patnaik BB, Tindwa H, Seo GW, Kim DH, Patnaik HH et al Genomic organization, sequence characterization and expression analysis of *Tenebrio molitor* apolipophorin-III in response to an intracellular pathogen. Gene. 534; 204–217. 10.1016/j.gene.2013.10.058 24200961

[81] Arancibia SA, Beltran CJ, Aguirre IM, Silva P, Peralta AL, Malinarich F et al Toll-like receptors are key participants in innate immune responses. Biol Res. 2007; 40: 97–112. 18064347 10.4067/s0716-97602007000200001

[82] Toubiana M, Rosani U, Giambelluca S, Cammarata M, Gerdol M, Pallavicini A et al Toll signal transduction pathway in bivalves: Complete cds of intermediate elements and related gene transcription levels in hemocytes of immune stimulated *Mytilus galloprovincialis*. Dev Comp Immunol. 2014; 300–312. 10.1016/j.dci.2014.03.021 24709052

[83] Tanguy M, McKenna P, Gauthier-Clerc S, Pellerin J, Danger J-M, Siah A. Sequence analysis of a normalized cDNA library of Mytilus edulis hemocytes exposed to Vibrio splendidus LGP32 strain. Results Immunol. 2013; 3: 40–50. 24600557 10.1016/j.rinim.2013.04.001 PMC3908323

[84] Zhang Y, He X, Yu F, Xiang Z, Li J, Thorpe KL et al Characteristic and functional analysis of Toll-like receptors (TLRs) in the lophotrochozoan, Crassostrea gigas, reveals ancient origin of TLR-mediated innate immunity. PLoS ONE. 8(10):e76464 10.1371/journal.pone.0076464 24098508 PMC3788107

[85] Ning X, Wang R, Li X, Wang S, Zhang M, Xing Q et al Genome-wide identification and characterization of five MyD88 duplication genes in Yesso scallop (Patinopecten yessoensis) and expression changes in response to bacterial challenge. Fish Shellfish Immunol. 2015. 10.1016/j.fsi.2015.06.028 26115632

[86] Guo X, He Y, Zhang L, Lelong C, Jouaux A. Immune and stress responses in oysters with insights on adaptation. Fish Shellfish Immunol. 2015; 46: 107–119. 10.1016/j.fsi.2015.05.018 25989624

[87] Zhang G, Fang X, Guo X, Li L, Luo R, Xu F et al The oyster genome reveals stress adaptation and complexity of shell formation. Nature. 2012; 490: 49–54. 10.1038/nature11413 22992520

[88] Diaz GA. Defensins and cysteine rich peptides: two types of antimicrobial peptides in marine molluscs. ISJ. 2010; 7: 157–164.

[89] Estari M, Satyanarayana J, Kumar BS, Bikshapati T, Reddy AS, Venkanna L. In vitro study of antimicrobial activity in freshwater mussel (Lamellidens marginalis) extract. Biol Med. 2011; 3: 191–195.

[90] Peng K, Wang J-H, Sheng J-Q, Zeng L-G, Hong Y-J. Molecular characterization and immune analysis of a defensin from freshwater pearl mussel, *Hyriopsis schlegelii*. Aquaculture. 2012; 334–337: 45–50. 10.1016/j.aquaculture.2011.12.039

[91] Ren Q, Li M, Zhang CY, Chen KP. Six defensins from the triangle-shell pearl mussel *Hyriopsis cumingii*. Fish Shellfish Immunol. 2011; 31: 1232–1238. 10.1016/j.fsi.2011.07.020 21839173

[92] Schmitt P, Wilmes M, Pugniere M, Aumelas A, Bachere E, Sahl HG et al Insight into invertebrate defensin mechanism of action: oyster defensins inhibit peptidoglycan biosynthesis by binding to lipid II. J Biol Chem. 2010; 285(38): 29208–29216. 10.1074/jbc.M110.143388 20605792 PMC2937951

[93] Roberts S, Goetz G, White S, Goetz F. Analysis of genes isolated from plated hemocytes of the Pacific oyster, Crassostrea gigas. Mar Biotech. 2009; 11: 24–44. 10.1007/s10126-008-9117-6 18622569

[94] Ma J, Zhang D, Jiang J, Cui S, Pu H, Jiang S. Molecular characterization and expression analysis of cathepsin L1 cysteine protease from pearl oyster *Pinctada fucata*. Fish Shellfish Immunol. 2010; 29: 501–507. 10.1016/j.fsi.2010.05.006 20573562

[95] Woo S, Denis V, Won H, Shin K, Lee G, Lee T-K et al Expressions of oxidative stress-related genes and antioxidant enzyme activities in Mytilus galloprovincialis (Bivalvia, Mollusca) exposed to hypoxia. Zool Studies. 2013; 52:15 10.1186/1810-522X-52-15

[96] Feder ME, Hofmann GE. Heat-shock proteins, molecular chaperones, and the stress response: evolutionary and ecological physiology. Annu Rev Physiol. 1999; 61: 243–282. 10099689 10.1146/annurev.physiol.61.1.243

[97] Zhang G, Fang X, Guo X, Li L, Luo R, Xu F et al The oyster genome reveals stress adaptation and complexity of shell formation. Nature. 2012; 490: 49–54. 10.1038/nature11413 22992520

[98] Zhang Z, Zhang Q. Molecular cloning, characterization and expression of heat shock protein 70 gene from the oyster Crassostrea hongkongensis responding to thermal stress and exposure of Cu(2+) and malachite green. Gene. 2012; 497(2): 172–180. 22310388 10.1016/j.gene.2012.01.058

[99] Leung PTY, Ip JCH, Mak SST, Qiu JW, Lam PKS, Wong CKC et al *De novo* transcriptome analysis of *Perna viridis* highlights tissue-specific patterns for environmental studies. BMC Genomics. 2014; 15:804 10.1186/1471-2164-15-804 25239240 PMC4190305

[100] Evans TG, Hofmann GE. Defining the limits of physiological plasticity: how gene expression can assess and predict the consequences of ocean change. Phil Trans R Soc B. 2012; 367: 1733–1745. 10.1098/rstb.2012.0019 22566679 PMC3350660

[101] Romero A, Estevez-Calvar N, Dios S, Figueras A, Novoa B. New insights into the apoptotic process in mollusks: characterization of caspase genes in Mytilus galloprovincialis. PLoS ONE. 2011; 6:e17003 10.1371/journal.pone.0017003 21347300 PMC3037946

[102] Venier P, Varotto L, Rosani U, Millino C, Celegato B, Bernante F et al Insights into the innate immunity of the Mediterranean mussel *Mytilus galloprovincialis*. BMC Genomics. 2011; 12:69 10.1186/1471-2164-12-69 21269501 PMC3039611

[103] Chavez-Villalba J, Soyez C, Huvet A, Gueguen Y, Lo C, Le Moullac G. Determination of gender in the pearl oyster *Pinctada margaritifera*. J Shellfish Res. 2011; 30: 231–240. 10.2983/035.030.0206

[104] Matsumoto T, Masaoka T, Fujiwara A, Nakamura Y, Satoh N, Awaji M. Reproduction-related genes in the pearl oyster genome. Zoological Sci. 2013; 30: 826–850. 10.2108/zsj.30.826 24125647

[105] Teaniniuraitemoana V, Huvet A, Levy P, Klopp C, Lhuillier E, Gaertner-Mazouni N et al Gonad transcriptome analysis of pearl oyster *Pinctada margaritifera*: identification of potential sex differentiation and sex determining genes. BMC Genomics. 2014; 15:491 10.1186/1471-2164-15-491 24942841 PMC4082630

[106] Naimi A, Martinez A-S, Specq M-L, Diss B, Mathieu M, Sourdaine P. Molecular cloning and gene expression of Cg-*Foxl2* during development and the adult gametogenetic cycle in the oyster *Crassostrea gigas*. Comp Biochem Physiol Part B. 2009; 154: 134–142. 10.1016/j.cbpb.2009.05.011 19481171

[107] Teaniniuraitemoana V, Huvet A, Levy P, Gaertner-Mazouni N, Gueguen Y, Moullac GL. Molecular signatures discriminating the male and the female sexual pathways in the pearl oyster Pinctada margaritifera. PLoS ONE. 2015; 10:e0122819 10.1371/journal.pone.0122819 25815473 PMC4376701

[108] Kopp A. *Dmrt* genes in the development and evolution of sexual dimorphism. Trends Genet. 2012; 28: 175–184. 10.1016/j.tig.2012.02.002 22425532 PMC3350790

[109] Lefebvre V, Dumitriu B, Penzo-Mendez A, Han Y, Pallavi B. Control of cell fate and differentiation by Sry-related high-mobility-group box (Sox) transcription factors. Int J Biochem Cell Biol. 2007; 39: 2195–2214. 17625949 10.1016/j.biocel.2007.05.019 PMC2080623

[110] Kashimada K, Koopman P. Sry: the master switch in mammalian sex determination. Development. 2010; 137: 3921–3930. 10.1242/dev.048983 21062860

[111] Tanaka SS, Nishinakamura R. Regulation of male sex determination: genital ridge formation and Sry activation in mice. Cell Mol Life Sci. 2014; 71: 4781–4802. 10.1007/s00018-014-1703-3 25139092 PMC4233110

[112] Oshima Y, Uno Y, Matsuda Y, Kobayashi T, Nakamura M. Molecular cloning and gene expression of *Foxl2* in the frog *Rana rugosa*. Gen Comp Endocrinol. 2008; 2–3: 170–177. 10.1016/j.ygcen.2008.08.013 18805419

[113] Yu FF, Wang MF, Zhou L, Gui JF, Yu XY. Molecular cloning and expression characterization of Dmrt2 in Akola Pearl Oysters, *Pinctada martensii*. J Shellfish Res. 2011; 30: 247–254.

[114] Liera-Herrera R, Garcia-Gasca A, Abreu-Goodger C, Huvet A, Ibarra AM. Identification of male gametogenesis expressed genes from the scallop Nodipecten subnodosus by suppressive subtraction hybridization and pyrosequencing. PLoS ONE. 2013; 8:e73176 10.1371/journal.pone.0073176 24066034 PMC3774672

[115] Matsumoto T, Nakamura AM, Mori K, Kayano T. Molecular characterization of a Cdna encoding putative vitellogenin from the pacific oyster Crassostrea gigas. Zoological Sci. 2003; 20: 37–42. 10.2108/zsj.20.37 12560599

[116] Osada M, Tawarayama H, Mori K. Estrogen synthesis in relation to gonadal development of Japanese scallop, *Pectinopecten yessoensis*: gonadal profile and immunolocalization of P450 aromatase and estrogen. Comp Biochem Physiol B. 2004; 139: 123–128. 15364295 10.1016/j.cbpc.2004.07.002

[117] Boutet I, Moraga D, Marinovic L, Obreque J, Chavez-Crooker P. Characterization of reproduction-specific genes in a marine bivalve mollusc: influence of maturation stage and sex on mRNA expression. Gene. 2008; 407: 130–138. 17976928 10.1016/j.gene.2007.10.005

[118] Li Y-C, Korol AB, Fahima T, Beiles A, Nevo E. Microsatellites: genomic distribution, putative functions and mutational mechanisms: a review. Mol Ecol. 2002; 11(12): 2453–2465. 10.1046/j.1365-294X.2002.01643.x 12453231

[119] Wang ZY, Fang BP, Chen JY, Zhang XJ, Luo ZX, Huang LF et al De novo assembly and characterization of root transcriptome using Illumina paired-end sequencing and development of cSSR markers in sweet potato (Ipomoea batatas). BMC Genomics. 2010, 11:726 10.1186/1471-2164-11-726 21182800 PMC3016421

[120] Abdul-Muneer PM. Application of microsatellite markers in conservation genetics and fisheries management: Recent advances in population structure analysis and conservation strategies. Genet Res Int. 2014, Article ID 691759. 10.1155/2014/691759 PMC3997932 24808959

[121] Wei G, Zhang L, Yan H, Zhao Y, Hu J, Pan W. Evaluation of the population structure and genetic diversity of *Plasmodium falciparum* in southern China. Malaria J. 2015; 14:283 10.1186/s12936-015-0786-0 PMC4509482 26194795

[122] Yan Y, Huang Y-L, Fang X, Lu L, Zhou R, Ge X-J et al Development and characterization of EST-SSR markers in the invasive weed Milkania micrantha (Asteraceae). Am J Bot. 2011; 98(1):E1–3. 10.3732/ajb.1000387 21613074

[123] Sanz N, Araguas RM, Vidal O, Diez-del-Molino D, Fernandez-Cebrian R, Garcia-Marin JL. Genetic characterization of the invasive mosquitofish (Gambusia spp.) introduced to Europe: population structure and colonization routes. Biol Invasions. 10.1007/s10530-013-0456-5

[124] Hoshino AA, Bravo JP, Macedo NP, Morelli KA. Microsatellites as tools for genetic diversity analysis. Genet Divers Microorganisms. 6: 149–170.

[125] Lopez-Uribe MM, Santiago CK, Bogdanowicz SM, Danforth BN. Discovery and characterization of microsatellites for the solitary bee *Colletes inaequalis* using Sanger and 454 pyrosequencing. Apidologie. 2013; 44: 163–172.

[126] Vidotto M, Grapputo A, Boscari E, Barbisan F, Coppe A, Grandi G et al Transcriptome sequencing and de novo annotation of the critically endangered Adriatic sturgeon. BMC Genomics. 2013; 14:407 10.1186/1471-2164-14-407 23773438 PMC3691660

[127] Penarrubia L, Araguas R-M, Pla C, Sanz N, Vinas J, Vidal O. Identification of 246 microsatellites in the Asiatic clam (Corbicula fluminea). Conservation Genet Resour. 2015; 7: 393–395.

[128] Jia ZY, Zhang YY, Shi LL, Bai QL, Jin SB, Mou ZB. Amplification of rainbow trout microsatellites in Brachymystax lenok (J). Mol Ecol Resour. 8(6): 1520–1521. 10.1111/j.1755-0998.2008.02310.x 21586095

[129] Ma K, Qiu G, Feng J, Li J. Transcriptome analysis of the oriental river prawn, Macrobrachium nipponense using 454 pyrosequencing for discovery of genes and markers. PLoS ONE. 7(6): e39727 10.1371/journal.pone.0039727 22745820 PMC3380025

