# Supplementary material for: Sequencing, De Novo Assembly, and Annotation of the Transcriptome of the Endangered Freshwater Pearl Bivalve, Cristaria plicata, Provides Novel Insights into Functional Genes and Marker Discovery
Source: PLoS One. 2016 Feb 12;11(2):e0148622. doi: 10.1371/journal.pone.0148622 (PMC4752248; doi:10.1371/journal.pone.0148622)
Supplement: S1 Table — (DOCX) [file pone.0148622.s001.docx]

**Supplementary Table-1**

Mapping of the assembled unigenes against *Cristaria plicata* mitochondrial protein coding genes using BLASTN at a cutoff E-value of 1E-5.

| Protein coding gene | Length (bp) | Sequence coverage (%) | Unigene ID |
| --- | --- | --- | --- |
| *NADH Dehydrogenase 5 (ND5)* | 1737 | 100 | 000887 |
| *Cytochrome C oxidase subunit I (COXI)* | 1542 | 100 |  |
| *NADH Dehydrogenase subunit 4 (ND4)* | 1347 | 100 |  |
| *Cytochrome B* | 1149 | 100 |  |
| *NADH Dehydrogenase subunit 1 (ND1)* | 900 | 100 |  |
| *Cytochrome C oxidase subunit III (COXIII)* | 780 | 100 |  |
| *ATP Synthase F0 subunit 6 (ATP6)* | 702 | 100 |  |
| *Cytochrome C oxidase subunit II (COXII)* | 681 | 100 |  |
| *NADH Dehydrogenase subunit 6 (ND6)* | 492 | 100 |  |
| *NADH Dehydrogenase subunit 3 (ND3)* | 357 | 100 |  |
| *NADH Dehydrogenase subunit 4L (ND4L)* | 297 | 100 |  |
| *ATP Synthase F0 subunit 8 (ATP8)* | 192 | 100 |  |
| *NADH Dehydrogenase subunit 2 (ND2)* | 964 | 100 | 009974 |
