# Supplementary material for: Sequencing, De Novo Assembly, and Annotation of the Transcriptome of the Endangered Freshwater Pearl Bivalve, Cristaria plicata, Provides Novel Insights into Functional Genes and Marker Discovery
Source: PLoS One. 2016 Feb 12;11(2):e0148622. doi: 10.1371/journal.pone.0148622 (PMC4752248; doi:10.1371/journal.pone.0148622)
Supplement: S3 Table — (DOCX) [file pone.0148622.s003.docx]

**S3 Table**

**Genes of interest for immune signaling response and defense mechanisms in *C. plicata* sequences**

| Candidate genes | *Unigene IDs* | *Length (bp)* |
| --- | --- | --- |
| **32 kDa beta-galactoside-binding lectin** | Cp_Uni_005290 | 250 |
| **chitinase-like lectin** | Cp_Uni_246177; Cp_Uni_246182 | 1240; 1767 |
| **chitin-binding lectin 1-like** | Cp_Uni_350172 | 1575 |
| **collectin-12-like** | Cp_Uni_044159 | 1325 |
| **C-type lectin** | Cp_Uni_002548; Cp_Uni_283355; Cp_Uni_014061; Cp_Uni_185512; Cp_Uni_049599; Cp_Uni_125810; Cp_Uni_091179; Cp_Uni_208107; Cp_Uni_091070; Cp_Uni_357848; Cp_Uni_002182; Cp_Uni_097821; Cp_Uni_215557; Cp_Uni_064686; Cp_Uni_035450; Cp_Uni_108779; Cp_Uni_175046; Cp_Uni_175051; Cp_Uni_175052; Cp_Uni_175054; Cp_Uni_215554; Cp_Uni_294259; Cp_Uni_230678; Cp_Uni_064813; Cp_Uni_030511; Cp_Uni_367030; Cp_Uni_280337; Cp_Uni_162615; Cp_Uni_169943; Cp_Uni_043660; Cp_Uni_004536; Cp_Uni_120471; Cp_Uni_346184; Cp_Uni_017360; Cp_Uni_044883; Cp_Uni_073014; Cp_Uni_317756 | 968; 354; 2041; 378; 426; 2463; 734; 944; 691; 435; 1043; 429; 1006; 386; 1135; 313; 359; 359; 283; 526; 752; 652; 1496; 697; 2751; 330; 547; 867; 699; 901; 627; 254; 251; 1366; 1749; 925; 468 |
| **endoplasmic reticulum lectin 1-like** | Cp_Uni_209045 | 3865 |
| **galectin** | Cp_Uni_062804; Cp_Uni_134152; Cp_Uni_057021; Cp_Uni_057022; Cp_Uni_065673; Cp_Uni_131186; Cp_Uni_131189; Cp_Uni_131191; Cp_Uni_150715; Cp_Uni_179303; Cp_Uni_179305; Cp_Uni_230000; Cp_Uni_230001; Cp_Uni_230002; Cp_Uni_230003; Cp_Uni_230004; Cp_Uni_230005; Cp_Uni_230006; Cp_Uni_230007; Cp_Uni_230008; Cp_Uni_230009; Cp_Uni_195481; Cp_Uni_220684; Cp_Uni_263037; Cp_Uni_019488; Cp_Uni_027505; Cp_Uni_004996 | 383; 299; 307; 291; 252; 624; 735; 391; 363; 440; 441; 1360; 1645; 1177; 1171; 1360; 2812; 1300; 1222; 862; 333; 617; 313; 246; 2477; 3003; 4818 |
| **E-selectin-like** | Cp_Uni_264899; Cp_Uni_007463; Cp_Uni_199732; Cp_Uni_199736; Cp_Uni_199737; Cp_Uni_359147; Cp_Uni_275809; Cp_Uni_275811 | 313; 938; 600; 866; 824; 369; 1555; 1256 |
| **F-type lectin 1** | Cp_Uni_118561 | 368 |
| **Fucolectin** | Cp_Uni_109273; Cp_Uni_246520; Cp_Uni_246521; Cp_Uni_246523; Cp_Uni_246524; Cp_Uni_246525; Cp_Uni_246529; Cp_Uni_246532; Cp_Uni_246535; Cp_Uni_086685; Cp_Uni_104287; Cp_Uni_175398; Cp_Uni_226111; Cp_Uni_252786; Cp_Uni_288172; Cp_Uni_175411; Cp_Uni_175417; Cp_Uni_209647; Cp_Uni_246526; Cp_Uni_246527; Cp_Uni_246531; Cp_Uni_246534; Cp_Uni_246537; Cp_Uni_246538; Cp_Uni_251900; Cp_Uni_251902; Cp_Uni_000208; Cp_Uni_142288; Cp_Uni_142289; Cp_Uni_003687; Cp_Uni_003688; Cp_Uni_233136; Cp_Uni_233137; Cp_Uni_248271; Cp_Uni_248272; Cp_Uni_248273; Cp_Uni_248274; Cp_Uni_248275; Cp_Uni_042194; Cp_Uni_081728; Cp_Uni_000207; Cp_Uni_209641; Cp_Uni_255935; Cp_Uni_175418; Cp_Uni_255926; Cp_Uni_000332; Cp_Uni_038733; Cp_Uni_251359; Cp_Uni_255930; Cp_Uni_255937; Cp_Uni_152078; Cp_Uni_177579; Cp_Uni_208981; Cp_Uni_208982; Cp_Uni_208983; Cp_Uni_208985; Cp_Uni_241280; Cp_Uni_241282; Cp_Uni_241286; Cp_Uni_241288; Cp_Uni_241289; Cp_Uni_241290; Cp_Uni_241292; Cp_Uni_241293; Cp_Uni_241294; Cp_Uni_241295; Cp_Uni_241296; Cp_Uni_241297; Cp_Uni_241298; Cp_Uni_245329; Cp_Uni_255922; Cp_Uni_116852; Cp_Uni_118563; Cp_Uni_118565; Cp_Uni_125369; Cp_Uni_255913; Cp_Uni_255927; Cp_Uni_255928; Cp_Uni_297382; Cp_Uni_255924; Cp_Uni_255925 | 598; 302; 978; 720; 1153; 1046; 1304; 1465; 1616; 238; 224; 555; 263; 314; 254; 712; 568; 869; 1543; 1270; 1681; 1832; 1307; 1392; 413; 446; 1510; 1096; 1522; 518; 768; 747; 654; 394; 394; 365; 418; 272; 293; 252; 1330; 1156; 388; 604; 278; 1415; 1050; 1085; 307; 305; 245; 677; 831; 1796; 1799; 578; 241; 815; 678; 1928; 550; 507; 747; 547; 1107; 1185; 1682; 830; 610; 227; 405; 586; 397; 345; 239; 347; 254; 230; 257; 768; 937 |
| **immulectin-3** | Cp_Uni_166032 | 441 |
| **I-type lectin-like protein 3** | Cp_Uni_027864 | 3923 |
| **lectin** | Cp_Uni_148642; Cp_Uni_243474 | 1373; 250 |
| **lectin subunit alpha-like** | Cp_Uni_185506; Cp_Uni_185507 | 517; 717 |
| **lectin-galC1** | Cp_Uni_294258 | 291 |
| **malectin-B-like** | Cp_Uni_215552; Cp_Uni_215553 | 770; 2029 |
| **mannan-binding lectin serine protease 2-like** | Cp_Uni_088260; Cp_Uni_088261; Cp_Uni_088262 | 2947; 2976; 363 |
| **sialic acid binding lectin** | Cp_Uni_000853; Cp_Uni_372460; Cp_Uni_070454 | 250; 305; 1558 |
| **tandem repeat galectin** | Cp_Uni_005291; Cp_Uni_005292; Cp_Uni_010304; Cp_Uni_081641; Cp_Uni_106424; Cp_Uni_128963; Cp_Uni_033401; Cp_Uni_037123; Cp_Uni_044418; Cp_Uni_115021; Cp_Uni_348839 | 471; 1859; 941; 616; 864; 1333; 450; 2788; 1771; 348; 2854 |
| **tachylectin-5A-like** | Cp_Uni_064569 | 1581 |
| **tachylectin-5B-like** | Cp_Uni_032326 | 2904 |
| **endothelial cells scavenger receptor** | Cp_Uni_090905; Cp_Uni_108865; Cp_Uni_108867; Cp_Uni_162731; Cp_Uni_162735; Cp_Uni_162736; Cp_Uni_162743; Cp_Uni_162744; Cp_Uni_162746; Cp_Uni_162747; Cp_Uni_208798; Cp_Uni_208992; Cp_Uni_040430; Cp_Uni_237905; Cp_Uni_012738; Cp_Uni_209017; Cp_Uni_209020; Cp_Uni_209021; Cp_Uni_209023; Cp_Uni_247377; Cp_Uni_247385 | 310; 392; 442; 381; 291; 267; 235; 348; 304; 293; 331; 280; 1174; 1339; 1517; 369; 255; 577; 761; 330; 510 |
| **scavenger receptor- Class A** | Cp_Uni_268298; Cp_Uni_323043 | 231; 479 |
| **scavenger receptor- Class B** | Cp_Uni_069555; Cp_Uni_172434; Cp_Uni_172435 | 1726; 2519; 2569 |
| **scavenger receptor- Class F** | Cp_Uni_263658; Cp_Uni_241278; Cp_Uni_162741; Cp_Uni_162742; Cp_Uni_162745; Cp_Uni_209663; Cp_Uni_209666; Cp_Uni_209670; Cp_Uni_247391; Cp_Uni_247396; Cp_Uni_209014; Cp_Uni_209654; Cp_Uni_071705; Cp_Uni_247400; Cp_Uni_214701; Cp_Uni_208998; Cp_Uni_209002; Cp_Uni_247373; Cp_Uni_247376; Cp_Uni_311326; Cp_Uni_209655; Cp_Uni_175416; Cp_Uni_175424; Cp_Uni_162737; Cp_Uni_019553; Cp_Uni_024222; Cp_Uni_046447; Cp_Uni_046448; Cp_Uni_100843; Cp_Uni_178165; Cp_Uni_208995; Cp_Uni_209652; Cp_Uni_247378; Cp_Uni_247395; Cp_Uni_162740; Cp_Uni_208802; Cp_Uni_069485; Cp_Uni_208991; Cp_Uni_005034 | 243; 231; 394; 398; 437; 1710; 978; 1711; 429; 324; 306; 342; 233; 321; 671; 406; 422; 276; 466; 314; 233; 683; 683; 258; 1065; 906; 329; 239; 409; 332; 436; 278; 244; 488; 294; 348; 268; 249; 433 |
| **scavenger receptor cysteine rich protein (SRCR)** | Cp_Uni_204533; Cp_Uni_355806; Cp_Uni_181995; Cp_Uni_108012; Cp_Uni_108013; Cp_Uni_325598; Cp_Uni_300019 | 1734; 390; 876; 1394; 1395; 256; 929 |
| **Lysozyme** | Cp_Uni_327043 | 238 |
| **lysozyme 1** | Cp_Uni_153007; Cp_Uni_013645; Cp_Uni_020324; Cp_Uni_035962; Cp_Uni_044105; Cp_Uni_054185; Cp_Uni_196773; Cp_Uni_231383; Cp_Uni_361304; Cp_Uni_349549; Cp_Uni_024121; Cp_Uni_348526 | 1776; 868; 2488; 1567; 2103; 576; 1064; 548; 807; 253; 751; 293 |
| **lysozyme 2** | Cp_Uni_030081; Cp_Uni_248590; Cp_Uni_036509; Cp_Uni_176289 | 2895; 2844; 1494; 735 |
| **lysozyme 3** | Cp_Uni_033181; Cp_Uni_041309; Cp_Uni_056259; Cp_Uni_098454 | 633; 850; 1370; 312 |
| **lysozyme precursor** | Cp_Uni_336455 | 465 |
| **phage-type lysozyme** | Cp_Uni_102540; Cp_Uni_144041; Cp_Uni_274554; Cp_Uni_361391 | 573; 987; 853; 231 |
| **putative GH family 25 lysozyme 4** | Cp_Uni_106932 | 283 |
| **Defensin** | Cp_Uni_004293; Cp_Uni_043733 | 552; 547 |
| **big defensin** | Cp_Uni_288695 | 1128 |
| **serpin B** | Cp_Uni_105827; Cp_Uni_105828; Cp_Uni_169290; Cp_Uni_169291; Cp_Uni_169292; Cp_Uni_169293; Cp_Uni_169294; Cp_Uni_235253 | 1090; 1069; 707; 1945; 1790; 1999; 1945; 314 |
| **alpha-2-macroglobulin** | Cp_Uni_144244; Cp_Uni_183735; Cp_Uni_275155; Cp_Uni_275158; Cp_Uni_275159; Cp_Uni_275161; Cp_Uni_002477; Cp_Uni_005689; Cp_Uni_008719; Cp_Uni_144604; Cp_Uni_144605; Cp_Uni_144606; Cp_Uni_144607; Cp_Uni_144608; Cp_Uni_189177; Cp_Uni_189178; Cp_Uni_189179; Cp_Uni_189180; Cp_Uni_267354; Cp_Uni_267355; Cp_Uni_275156; Cp_Uni_275157; Cp_Uni_275160; Cp_Uni_370937; Cp_Uni_370938 | 447; 937; 2368; 2008; 2047; 243; 4572; 1465; 5743; 540; 556; 520; 576; 556; 1610; 1565; 1608; 1670; 303; 303; 964; 1138; 610; 265; 265 |
| **astacin-like** | Cp_Uni_220401; Cp_Uni_021190 | 1456; 1530 |
| **toll-like receptor 13** | Cp_Uni_283773; Cp_Uni_015312; Cp_Uni_032299; Cp_Uni_038286; Cp_Uni_003041; Cp_Uni_022025; Cp_Uni_042404; Cp_Uni_229165; Cp_Uni_190877; Cp_Uni_190878 | 659; 954; 2747; 714; 1902; 2756; 1179; 507; 769; 579 |
| **toll-like receptor 2** | Cp_Uni_110644; Cp_Uni_192893; Cp_Uni_037564; Cp_Uni_110645; Cp_Uni_121146; Cp_Uni_163539; Cp_Uni_163540; Cp_Uni_006588; Cp_Uni_192891; Cp_Uni_020706; Cp_Uni_064037; Cp_Uni_147194; Cp_Uni_192884; Cp_Uni_192885; Cp_Uni_192886; Cp_Uni_192887; Cp_Uni_192889; Cp_Uni_192890; Cp_Uni_192892; Cp_Uni_282338; Cp_Uni_192894; Cp_Uni_129115; Cp_Uni_202821; Cp_Uni_001086; Cp_Uni_001088; Cp_Uni_001089; Cp_Uni_005707; Cp_Uni_020329; Cp_Uni_115878; Cp_Uni_115880; Cp_Uni_115881; Cp_Uni_123143; Cp_Uni_123144; Cp_Uni_123146; Cp_Uni_123147; Cp_Uni_123148; Cp_Uni_123151; Cp_Uni_160135; Cp_Uni_160136; Cp_Uni_160139; Cp_Uni_185283; Cp_Uni_185285; Cp_Uni_185286; Cp_Uni_233542; Cp_Uni_235990; Cp_Uni_235991; Cp_Uni_235993; Cp_Uni_241173; Cp_Uni_241177; Cp_Uni_241178; Cp_Uni_241179; Cp_Uni_241180; Cp_Uni_241183; Cp_Uni_241184; Cp_Uni_241185; Cp_Uni_246310; Cp_Uni_246311; Cp_Uni_248192; Cp_Uni_248193; Cp_Uni_248194; Cp_Uni_248196; Cp_Uni_248197; Cp_Uni_248198; Cp_Uni_248199; Cp_Uni_248200; Cp_Uni_265882; Cp_Uni_265883; Cp_Uni_275033; Cp_Uni_359113 | 767; 420; 1661; 701; 2444; 2922; 2976; 2545; 741; 1442; 804; 1640; 800; 741; 1997; 1985; 2393; 1776; 1997; 479; 2509; 477; 734; 3336; 1950; 3397; 1584; 2765; 2175; 2214; 1632; 2228; 1247; 1490; 712; 2338; 2350; 1858; 2275; 1786; 3026; 2714; 4084; 1042; 384; 560; 884; 588; 485; 654; 984; 1189; 359; 469; 1475; 1836; 1785; 308; 308; 308; 272; 470; 246; 329; 329; 335; 349; 1881; 384 |
| **toll-like receptor 3** | Cp_Uni_209417; Cp_Uni_041115 | 227; 1079 |
| **toll-like receptor 4** | Cp_Uni_283768; Cp_Uni_283770; Cp_Uni_283771; Cp_Uni_283772; Cp_Uni_283775; Cp_Uni_021366; Cp_Uni_038940; Cp_Uni_199343; Cp_Uni_266664; Cp_Uni_266665; Cp_Uni_266666; Cp_Uni_266667; Cp_Uni_266668; Cp_Uni_122975; Cp_Uni_122976; Cp_Uni_122979; Cp_Uni_192888; Cp_Uni_045391; Cp_Uni_188211; Cp_Uni_119035; Cp_Uni_119036; Cp_Uni_192895; Cp_Uni_192896; Cp_Uni_004278; Cp_Uni_358170; Cp_Uni_020835; Cp_Uni_062700; Cp_Uni_004439; Cp_Uni_204539 | 947; 999; 549; 643; 963; 1898; 1852; 922; 2218; 1828; 1788; 2315; 450; 3479; 4020; 4769; 926; 1108; 1644; 2123; 2115; 993; 820; 2800; 409; 4390; 486; 2419;1936 |
| **toll-like receptor 6** | Cp_Uni_017807 | 379 |
| **toll-like receptor 7** | Cp_Uni_147195 | 250 |
| **toll-like receptor c** | Cp_Uni_000140; Cp_Uni_013854; Cp_Uni_020301; Cp_Uni_129116; Cp_Uni_209424; Cp_Uni_209425; Cp_Uni_209426 | 5108; 1599; 3175; 363; 3106; 2893; 2098 |
| **toll-like receptor e precursor** | Cp_Uni_010844; Cp_Uni_040989; Cp_Uni_147193; Cp_Uni_190223; Cp_Uni_339758 | 3533; 3597; 898; 641; 588; |
| **toll-like receptor f precursor** | Cp_Uni_005650; Cp_Uni_012960; Cp_Uni_030953; Cp_Uni_119037; Cp_Uni_132132; Cp_Uni_262167; Cp_Uni_273542 | 3098; 2063; 1954; 457; 415; 3704; 1205 |
| **toll-like receptor g precursor** | Cp_Uni_000032; Cp_Uni_019453; Cp_Uni_034542; Cp_Uni_040373; Cp_Uni_040390; Cp_Uni_047330; Cp_Uni_149554; Cp_Uni_152007; Cp_Uni_152008; Cp_Uni_152010; Cp_Uni_282340; Cp_Uni_282342; Cp_Uni_282343; Cp_Uni_283774 | 1479; 2187; 351; 1284; 1297; 240; 1100; 2868; 2732; 2727; 701; 711; 645; 632 |
| **toll-like receptor h precursor** | Cp_Uni_149244 | 368 |
| **toll-like receptor i** | Cp_Uni_006633; Cp_Uni_013221; Cp_Uni_087506; Cp_Uni_145405 | 2960; 1012; 358; 1407 |
| **toll-like receptor k** | Cp_Uni_282566; Cp_Uni_290675; Cp_Uni_290676; Cp_Uni_290677 | 406; 5129; 4907; 4942 |
| **toll-like receptor m** | Cp_Uni_000022; Cp_Uni_000028; Cp_Uni_000029; Cp_Uni_000031; Cp_Uni_002874; Cp_Uni_002875; Cp_Uni_019983; Cp_Uni_023520; Cp_Uni_040456; Cp_Uni_110643; Cp_Uni_158818; Cp_Uni_268816; Cp_Uni_273541; Cp_Uni_282339; Cp_Uni_362214 | 836; 1487; 496; 1179; 3181; 5060; 3366; 3301; 303; 344; 1694; 530; 1714; 377; 401 |
| **toll-like receptor R precursor** | Cp_Uni_282341 | 510 |
| **toll-like receptor S precursor** | Cp_Uni_008473; Cp_Uni_197567; Cp_Uni_209423 | 2344; 439; 2311 |
| **toll-like receptor W precursor** | Cp_Uni_131432 | 301 |
| **MYD88** | Cp_Uni_026390 | 1790 |
| **IRAK1 protein** | Cp_Uni_268172; Cp_Uni_268181; Cp_Uni_268183 | 2045; 2801; 1961 |
| **relish** | Cp_Uni_163814 | 5704 |
| **c-Jun N-terminal kinase JNK** | Cp_Uni_154142; Cp_Uni_154143; Cp_Uni_154144 | 4540; 3657; 4293 |
| **AP-1 protein** | Cp_Uni_299471 | 1576 |
| **protein pellino-like** | Cp_Uni_004695 | 7530 |
| **caspase** | Cp_Uni_068989; Cp_Uni_068990; Cp_Uni_068991 | 348; 344; 349 |
| **caspase 3/7-3** | Cp_Uni_044543; Cp_Uni_088973; Cp_Uni_232273 | 3783; 243; 1542 |
| **caspase 3-like protein** | Cp_Uni_103152 | 1429 |
| **caspase activity and apoptosis inhibitor 1-like isoform X1** | Cp_Uni_133884; Cp_Uni_133890; Cp_Uni_133891; Cp_Uni_133892 | 2727; 2366; 2103; 1985 |
| **caspase recruitment domain protein** | Cp_Uni_221910; Cp_Uni_221913; Cp_Uni_020593; Cp_Uni_189168; Cp_Uni_189169; Cp_Uni_224187; Cp_Uni_366924; Cp_Uni_217041; Cp_Uni_217042; Cp_Uni_217043 | 268; 267; 1070; 1296; 1334; 665; 772; 6145; 6223; 5968 |
| **caspase-1** | Cp_Uni_112529; Cp_Uni_273293; Cp_Uni_273297; Cp_Uni_232272; Cp_Uni_004476; Cp_Uni_010133; Cp_Uni_251682; Cp_Uni_251685; Cp_Uni_273061; Cp_Uni_273063; Cp_Uni_273064; Cp_Uni_251683; Cp_Uni_251684; Cp_Uni_251686; Cp_Uni_261183 | 1450; 1726; 630; 2326; 2640; 4677; 1924; 1293; 2170; 2313; 1469; 2086; 1421; 1917; 842 |
| **caspase-2** | Cp_Uni_255100; Cp_Uni_026770; Cp_Uni_031781; Cp_Uni_111616 | 3186; 1826; 1561; 1476 |
| **caspase-3** | Cp_Uni_216322; Cp_Uni_228861; Cp_Uni_094162; Cp_Uni_094163; Cp_Uni_261180; Cp_Uni_261181; Cp_Uni_261182; Cp_Uni_005893; Cp_Uni_273065; Cp_Uni_050244; | 2327; 2747; 1956; 1185; 1898; 1795; 1860; 2510; 1974; 1341 |
| **caspase-7** | Cp_Uni_127800; Cp_Uni_032712; Cp_Uni_005572; Cp_Uni_273291; Cp_Uni_273292; Cp_Uni_273294; Cp_Uni_273295; Cp_Uni_273296 | 770; 3050; 3214; 2138; 2021; 1198; 1890; 1042 |
| **caspase-8** | Cp_Uni_008628; Cp_Uni_216303; Cp_Uni_216327; Cp_Uni_005927; Cp_Uni_020347; Cp_Uni_025464; Cp_Uni_181048 | 2144; 1080; 2065; 1657; 1988; 3950; 1965 |
| **caspase-9-like isoform X2** | Cp_Uni_232966 | 1029 |
| **caspase-10** | Cp_Uni_007148; Cp_Uni_031484; Cp_Uni_232271; Cp_Uni_232274; Cp_Uni_251681 | 3859; 2435; 2692; 1388; 841 |
| **putative caspase, partial** | Cp_Uni_273066 | 1743 |
| **Bcl-2** | Cp_Uni_209734; Cp_Uni_100453; Cp_Uni_329938; Cp_Uni_360713; Cp_Uni_095552; Cp_Uni_339501; Cp_Uni_274171; Cp_Uni_274172; Cp_Uni_274173; Cp_Uni_274174 | 4851; 1808; 954; 1768; 3316; 281; 3277; 3355; 3318; 3240 |
| **BAX** | Cp_Uni_014002 | 1713 |
| **cathepsin B** | Cp_Uni_363642; Cp_Uni_289455; Cp_Uni_372462; Cp_Uni_278644; Cp_Uni_053764; Cp_Uni_261810; Cp_Uni_034048; Cp_Uni_308646; Cp_Uni_126491; Cp_Uni_146947; Cp_Uni_176261; Cp_Uni_310090; Cp_Uni_113636; Cp_Uni_254 | 247; 1910; 383; 253; 315; 455; 448; 329; 568; 443; 347; 277; 229; 493 |
| **cathepsin C** | Cp_Uni_166248; Cp_Uni_166249; Cp_Uni_166250 | 1797; 1776; 2482 |
| **cathepsin D** | Cp_Uni_098645; Cp_Uni_312637; Cp_Uni_070568 | 1488; 255; 693 |
| **cathepsin F** | Cp_Uni_174808; Cp_Uni_174809; Cp_Uni_174810; Cp_Uni_174812; Cp_Uni_174813; Cp_Uni_174815; Cp_Uni_174816; Cp_Uni_174818; Cp_Uni_174819 | 3069; 3409; 2949; 2815; 2887; 2794; 5165; 2863; 2949 |
| **cathepsin L** | Cp_Uni_184601; Cp_Uni_097638; Cp_Uni_306846; Cp_Uni_195337; Cp_Uni_034338; Cp_Uni_013810; Cp_Uni_024561; Cp_Uni_099685; Cp_Uni_099686; Cp_Uni_099687; Cp_Uni_145995; Cp_Uni_145996; Cp_Uni_145997; Cp_Uni_145998; Cp_Uni_145999; Cp_Uni_345011; Cp_Uni_176311; Cp_Uni_308792; Cp_Uni_012116; Cp_Uni_051984; Cp_Uni_182735; Cp_Uni_190689; Cp_Uni_168336; Cp_Uni_111776; Cp_Uni_111778; Cp_Uni_111779; Cp_Uni_163252; Cp_Uni_367162; Cp_Uni_367163; Cp_Uni_198039; Cp_Uni_198040; Cp_Uni_198041; Cp_Uni_198042; Cp_Uni_162933; Cp_Uni_134337; Cp_Uni_354673; Cp_Uni_163264; Cp_Uni_321616; Cp_Uni_004468; Cp_Uni_064397; Cp_Uni_120861; Cp_Uni_196427; Cp_Uni_163260; Cp_Uni_083268; Cp_Uni_293838; Cp_Uni_140154; Cp_Uni_205893; Cp_Uni_023824; Cp_Uni_025731; Cp_Uni_010756; Cp_Uni_039808; Cp_Uni_059974; Cp_Uni_122750; Cp_Uni_122751; Cp_Uni_122752; Cp_Uni_163261; Cp_Uni_163262; Cp_Uni_258450; Cp_Uni_013845; Cp_Uni_082715; Cp_Uni_163253; Cp_Uni_163263; Cp_Uni_062340; Cp_Uni_062356; Cp_Uni_068208; Cp_Uni_000453; Cp_Uni_000455; Cp_Uni_000456; Cp_Uni_000457; Cp_Uni_010561; Cp_Uni_023111; Cp_Uni_025457; Cp_Uni_035109; Cp_Uni_041503; Cp_Uni_042770; Cp_Uni_069658; Cp_Uni_078664; Cp_Uni_078665; Cp_Uni_097728; Cp_Uni_153872; Cp_Uni_179455; Cp_Uni_205894; Cp_Uni_205895; Cp_Uni_205896; Cp_Uni_205897; Cp_Uni_205898; Cp_Uni_205899; Cp_Uni_205900; Cp_Uni_205901; Cp_Uni_205902; Cp_Uni_205903; Cp_Uni_205904; Cp_Uni_205905; Cp_Uni_205906; Cp_Uni_205907; Cp_Uni_215371; Cp_Uni_364245; Cp_Uni_168882; Cp_Uni_044194; Cp_Uni_080162; Cp_Uni_009162; Cp_Uni_238530; Cp_Uni_168335; Cp_Uni_329227; Cp_Uni_168337; Cp_Uni_198038; Cp_Uni_163254; Cp_Uni_163256; Cp_Uni_163258; Cp_Uni_349692; Cp_Uni_149392; Cp_Uni_062147; Cp_Uni_300451; Cp_Uni_348136; Cp_Uni_163255; Cp_Uni_163257; Cp_Uni_163259 | 399; 388; 307; 282; 454; 305; 619; 265; 289; 358; 1390; 1393; 1302; 1390; 449; 319; 578; 395; 1019; 869; 361; 616; 504; 245; 408; 436; 228; 409; 409; 771; 775; 730; 730; 319; 441; 455; 248; 282; 1774; 570; 390; 320; 224; 534; 1544; 703; 552; 3199; 610; 1966; 1563; 321; 2403; 2463; 2420; 544; 544; 227; 2167; 311; 292; 626; 1313; 256; 884; 1321; 1032; 400; 905; 406; 892; 824; 809; 283; 1724; 268; 485; 298; 386; 592; 387; 322; 380; 544; 2108; 578; 323; 2107; 1599; 973; 380; 1362; 1191; 882; 457; 1334; 227; 1146; 932; 278; 575; 1657; 460; 227; 355; 467; 502; 304; 437; 301; 313; 514; 405; 336; 576; 607; 393 |
| **cathepsin S, partial** | Cp_Uni_128383; Cp_Uni_271672; Cp_Uni_271673; Cp_Uni_271674; Cp_Uni_271676; Cp_Uni_271677; Cp_Uni_271678; Cp_Uni_271679; Cp_Uni_271680; Cp_Uni_271681 | 247; 231; 255; 231; 231; 673; 565; 1181; 1160; 255 |
| **cathepsin Z-like** | Cp_Uni_225722 | 3195 |
| **glutathione peroxidase** | Cp_Uni_000475; Cp_Uni_157082; Cp_Uni_218008; Cp_Uni_218009; Cp_Uni_218010; Cp_Uni_218011; Cp_Uni_218012; Cp_Uni_218013; Cp_Uni_218015; Cp_Uni_218016; Cp_Uni_218017; Cp_Uni_218018; Cp_Uni_218021; Cp_Uni_218022; Cp_Uni_218024; Cp_Uni_218025; Cp_Uni_218026; Cp_Uni_218027; Cp_Uni_218029; Cp_Uni_218030; Cp_Uni_218031; Cp_Uni_218032; Cp_Uni_218034; Cp_Uni_218035; Cp_Uni_218036; Cp_Uni_218037; Cp_Uni_218038; Cp_Uni_218039; Cp_Uni_035987; Cp_Uni_313925; Cp_Uni_000472; Cp_Uni_000473; Cp_Uni_000476; Cp_Uni_165140; Cp_Uni_275431; Cp_Uni_354675; Cp_Uni_007384; Cp_Uni_042911; Cp_Uni_358203 | 664; 750; 1052; 379; 387; 369; 381; 383; 375; 258; 383; 377; 385; 389; 385; 377; 371; 373; 381; 373; 375; 379; 387; 371; 393; 369; 391; 262; 879; 894; 2047; 781; 3992; 1178; 1401; 526; 4563; 1327; 252 |
| **Mn-superoxide dismutase(SOD)** | Cp_Uni_263014 | 1112 |
| **Cu-Zn-superoxide dismutase(SOD)** | Cp_Uni_025370; Cp_Uni_275444; Cp_Uni_306390; Cp_Uni_236458; Cp_Uni_091557; Cp_Uni_016474; Cp_Uni_110031; Cp_Uni_063687; Cp_Uni_085441; Cp_Uni_038336 | 1449; 547; 388; 776; 2382; 895; 326; 1051; 480; 1462 |
| **glutathione S-transferase** | Cp_Uni_353418; Cp_Uni_302450; Cp_Uni_342684; Cp_Uni_074703; Cp_Uni_209373; Cp_Uni_347183; Cp_Uni_010947; Cp_Uni_182839; Cp_Uni_220414; Cp_Uni_165252 | 230; 319; 512; 1312; 1030; 718; 1021; 447; 504; 1217 |
| **glutathione S-transferase 1** | Cp_Uni_189600; Cp_Uni_022625; Cp_Uni_199185 | 317; 2177; 276 |
| **glutathione S-transferase alpha** | Cp_Uni_046399; Cp_Uni_046400 | 2010; 1940 |
| **glutathione S-transferase C-terminal domain-containing protein** | Cp_Uni_206477; Cp_Uni_206478; Cp_Uni_206475; Cp_Uni_206476 | 303; 1785; 2824; 2597 |
| **glutathione s-transferase M2** | Cp_Uni_204657 | 298 |
| **glutathione S-transferase mu** | Cp_Uni_338960; Cp_Uni_370546; Cp_Uni_004445; Cp_Uni_257229 | 255; 439; 1646; 1822 |
| **glutathione S-transferase omega-1-like** | Cp_Uni_341225; Cp_Uni_280553; Cp_Uni_280554; Cp_Uni_280555; Cp_Uni_280556; Cp_Uni_280557 | 986; 2180; 2541; 2137; 2525; 2568 |
| **glutathione S-transferase P2** | Cp_Uni_073612 | 1353 |
| **glutathione S-transferase pi** | Cp_Uni_054731; Cp_Uni_225678; Cp_Uni_030371; Cp_Uni_366042 | 234; 1012; 901; 279 |
| **glutathione S-transferase sigma** | Cp_Uni_115274; Cp_Uni_121625; Cp_Uni_150519; Cp_Uni_238577; Cp_Uni_238578; Cp_Uni_333851; Cp_Uni_093816; Cp_Uni_093817; Cp_Uni_094446; Cp_Uni_307107; Cp_Uni_339151 | 1155; 491; 315; 996; 1127; 912; 1650; 1441; 1804; 1550; 1099 |
| **glutathione S-transferase theta** | Cp_Uni_088581 | 521 |
| **microsomal glutathione S-transferase** | Cp_Uni_009853; Cp_Uni_041930; Cp_Uni_120171; Cp_Uni_078095; Cp_Uni_078096; Cp_Uni_333894; Cp_Uni_088844 | 1354; 488; 1274; 941; 1295; 879; 1053 |
| **glutathione synthetase** | Cp_Uni_192993; Cp_Uni_192992 | 728; 4338 |
| **catalase** | Cp_Uni_243973; Cp_Uni_324094; Cp_Uni_053910; Cp_Uni_001142; Cp_Uni_001145; Cp_Uni_023789; Cp_Uni_169470; Cp_Uni_311083; Cp_Uni_264001; Cp_Uni_036148; Cp_Uni_204444 | 343; 1337; 248; 2668; 1387; 1702; 1125; 271; 274; 4628; 490 |
| **apolipophorin** | Cp_Uni_008431; Cp_Uni_008678; Cp_Uni_155477; Cp_Uni_292283; Cp_Uni_346491; Cp_Uni_070163; Cp_Uni_317274 | 14125; 1504; 498; 363; 316; 257; 380 |
| **peptidoglycan recognition protein 1** | Cp_Uni_022723; Cp_Uni_025707 | 322; 1626 |
| **peptidoglycan recognition protein 2** | Cp_Uni_024500 | 1286 |
| **peptidoglycan recognition protein S1** | Cp_Uni_003948; Cp_Uni_003949; Cp_Uni_003950; Cp_Uni_114960; Cp_Uni_162662; Cp_Uni_16266 | 951; 296; 1209; 238; 1426; 224 |
| **peptidoglycan recognition protein S2** | Cp_Uni_037409; Cp_Uni_146316; Cp_Uni_347900 | 570; 2652; 1253 |
| **peptidoglycan recognition protein long** | Cp_Uni_215750 | 2155 |
| **peptidoglycan recognition protein short** | Cp_Uni_012851 | 2862 |
| **peptidoglycan-recognition protein, partial** | Cp_Uni_361283 | 652 |
| **CD63 antigen** | Cp_Uni_282882; Cp_Uni_035432; Cp_Uni_005868; Cp_Uni_139911; Cp_Uni_174931; Cp_Uni_026032; Cp_Uni_258606; Cp_Uni_002221; Cp_Uni_123137 | 314; 2496; 1219; 661; 859; 1867; 3831; 3334; 1702 |
| **thioester-containing protein** | Cp_Uni_321678; Cp_Uni_009114; Cp_Uni_094175; Cp_Uni_164527; Cp_Uni_296081; Cp_Uni_264470; Cp_Uni_102548; Cp_Uni_134683 | 324; 833; 333; 341; 346; 277; 245; 224 |
| **complement C1q** | Cp_Uni_292249; Cp_Uni_198335; Cp_Uni_038660; Cp_Uni_220462; Cp_Uni_011323; Cp_Uni_051996; Cp_Uni_023770; Cp_Uni_118650; Cp_Uni_118652; Cp_Uni_000850; Cp_Uni_000851; Cp_Uni_000852; Cp_Uni_000854; Cp_Uni_000855; Cp_Uni_042200; Cp_Uni_118651; Cp_Uni_142375; Cp_Uni_020992; Cp_Uni_235068; Cp_Uni_235069; Cp_Uni_019898; Cp_Uni_316571; Cp_Uni_011611; Cp_Uni_223522; Cp_Uni_013510; Cp_Uni_043355; Cp_Uni_045725; Cp_Uni_344695; Cp_Uni_259333; Cp_Uni_038234; Cp_Uni_170901; Cp_Uni_304491; Cp_Uni_037038; Cp_Uni_164949; Cp_Uni_202264; Cp_Uni_009874; Cp_Uni_175015; Cp_Uni_046350; Cp_Uni_305868; Cp_Uni_330148; Cp_Uni_361755; Cp_Uni_145627; Cp_Uni_281928; Cp_Uni_095356; Cp_Uni_122918; Cp_Uni_088634; Cp_Uni_163899; Cp_Uni_170854; Cp_Uni_170857; Cp_Uni_305362; Cp_Uni_031475; Cp_Uni_009287; Cp_Uni_373768; Cp_Uni_033158; Cp_Uni_066713; Cp_Uni_066714; Cp_Uni_199377; Cp_Uni_306393; Cp_Uni_316381; Cp_Uni_088643; Cp_Uni_023212; Cp_Uni_038886; Cp_Uni_304744; Cp_Uni_348814; Cp_Uni_009730; Cp_Uni_351052; Cp_Uni_170856; Cp_Uni_038397; Cp_Uni_128629; Cp_Uni_214103; Cp_Uni_013061; Cp_Uni_008639; Cp_Uni_049942; Cp_Uni_031217; Cp_Uni_353546; Cp_Uni_348828; Cp_Uni_034405; Cp_Uni_163897; Cp_Uni_204641; Cp_Uni_040471; Cp_Uni_228231; Cp_Uni_186768 | 1676; 1771; 1444; 768; 1211; 1031; 2010; 331; 417; 1143; 987; 329; 1135; 1881; 450; 256; 619; 1141; 2089; 3267; 3535; 899; 1326; 880; 1484; 999; 1358; 918; 246; 1164; 1352; 1470; 529; 375; 1476; 3688; 908; 2332; 1159; 1139; 347; 592; 390; 964; 1354; 1713; 1871; 1190; 1095; 1503; 1257; 2147; 299; 791; 869; 1092; 1047; 804; 905; 391; 1844; 1524; 367; 1598; 1284; 1973; 1119; 3332; 1004; 1202; 1411; 666; 471; 1059; 268; 894; 1328; 699; 319; 667; 345; 226 |
| **p38** | Cp_Uni_153427 | 1146 |
| **heat shock protein** | Cp_Uni_152543; Cp_Uni_113276; Cp_Uni_317561; Cp_Uni_134587; Cp_Uni_160365; Cp_Uni_357812; Cp_Uni_316872 | 277; 2096; 253; 339; 245; 518; 351 |
| **small heat shock protein** | Cp_Uni_169727; Cp_Uni_038695 | 1377; 1049 |
| **heat shock protein 10** | Cp_Uni_238625 | 1044 |
| **heat shock protein 12** | Cp_Uni_081773; Cp_Uni_062608; Cp_Uni_127463 | 235; 339; 301 |
| **heat shock protein 20** | Cp_Uni_097082; Cp_Uni_097083; Cp_Uni_089289 | 1226; 1567; 2263 |
| **heat shock protein 40** | Cp_Uni_167624 | 256 |
| **heat shock protein 60** | Cp_Uni_298637; Cp_Uni_323782; Cp_Uni_121261 | 242; 329; 2343 |
| **heat shock protein 67B2** | Cp_Uni_125049 | 5278 |
| **heat shock protein 70** | Cp_Uni_069146; Cp_Uni_254497; Cp_Uni_282410; Cp_Uni_282411; Cp_Uni_273509; Cp_Uni_256824; Cp_Uni_291504; Cp_Uni_291519; Cp_Uni_291523; Cp_Uni_291532; Cp_Uni_291533; Cp_Uni_291534; Cp_Uni_371872; Cp_Uni_371873; Cp_Uni_035323; Cp_Uni_038718 | 442; 241; 1180; 1239; 2662; 2967; 529; 584; 479; 1865; 531; 482; 578; 255; 447; 503 |
| **heat shock protein 75** | Cp_Uni_368197 | 3099 |
| **heat shock protein 78** | Cp_Uni_291009 | 4125 |
| **heat shock protein 90** | Cp_Uni_120859; Cp_Uni_280311; Cp_Uni_322805; Cp_Uni_024740; Cp_Uni_059977; Cp_Uni_152587; Cp_Uni_152588; Cp_Uni_105518; Cp_Uni_066432; Cp_Uni_271180; Cp_Uni_308486; Cp_Uni_124475 | 2639; 500; 294; 2930; 490; 586; 515; 1941; 273; 398; 374; 462 |
| **heat shock protein cognate 3** | Cp_Uni_315613 | 551 |
| **heat shock protein cognate 5** | Cp_Uni_367500; Cp_Uni_326472 | 322; 328 |
| **peroxiredoxin** | Cp_Uni_355803; Cp_Uni_222962; Cp_Uni_323498; Cp_Uni_342966; Cp_Uni_365428; Cp_Uni_126349; Cp_Uni_126350; Cp_Uni_126351; Cp_Uni_055605 | 1772; 429; 330; 324; 1131; 1444; 1004; 509; 3675 |
