# Supplementary material for: Sequencing, De Novo Assembly, and Annotation of the Transcriptome of the Endangered Freshwater Pearl Bivalve, Cristaria plicata, Provides Novel Insights into Functional Genes and Marker Discovery
Source: PLoS One. 2016 Feb 12;11(2):e0148622. doi: 10.1371/journal.pone.0148622 (PMC4752248; doi:10.1371/journal.pone.0148622)
Supplement: S4 Table — (DOCX) [file pone.0148622.s004.docx]

S4 Table: Candidate genes for sex-determination and reproduction in *C. plicata* transcriptome

| Protein family | Unigene ID | Length (bp) |
| --- | --- | --- |
| Sex-determination and differentiation related | | |
| WD repeat and HMG-box DNA-binding protein 1 | Cp_Uni_029636 | 4196 |
| HMG-box transcriptional regulator | Cp_Uni_157480 | 365 |
| Wnt 4 protein | Cp_Uni_096208 | 1658 |
| Steroidogenic factor-1 like isoform | Cp_Uni_165678; Cp_Uni_165679; Cp_Uni_282653 | 4483; 4443; 3650 |
| DAX1 | Cp_Uni_022940 | 2246 |
| SOX-5 | Cp_Uni_004071; Cp_Uni_004072; Cp_Uni_004073; Cp_Uni_004074 | 3530; 3150; 3253; 3639 |
| SOX-6 | Cp_Uni_157889 | 393 |
| SOX-9 | Cp_Uni_157481 | 4159 |
| SOX-11 | Cp_Uni_318595 | 2273 |
| SOX-15 | Cp_Uni_297074 | 2396 |
| SOXB2 | Cp_Uni_020182 | 1633 |
| DMRT1-testis specific | Cp_Uni_233275 | 1414 |
| Doublesex and mab-3 related transcription factor | Cp_Uni_366611; Cp_Uni_044057; Cp_Uni_070130; Cp_Uni_025914; Cp_Uni_238157; Cp_Uni_238158; Cp_Uni_302545; Cp_Uni_279873;  Cp_Uni_279874; Cp_Uni_279875 | 1489; 2418; 2273; 1790; 1781; 1844; 1641; 1053; 872; 739 |
| Sex comb on midleg-like protein 2 | Cp_Uni_083210 | 3596 |
| Complementary sex determiner | Cp_Uni_198143; Cp_Uni_198144 | 555; 579 |
| WNT | Cp_Uni_232160; Cp_Uni_063623; Cp_Uni_112457; Cp_Uni_292411; Cp_Uni_019431; Cp_Uni_016481; Cp_Uni_096208; Cp_Uni_130353; Cp_Uni_130354; Cp_Uni_020943; Cp_Uni_293849; Cp_Uni_148172; Cp_Uni_043153; | 2211; 1529; 1698; 2188; 2950; 3597; 1658; 1720; 1553; 2792; 5476; 3465; 1028; |
| Sex determining protein Fem-1 like protein | Cp_Uni_013399 | 2058 |
| Sex determining region Y protein-like isoform | Cp_Uni_001001 | 621 |
| Reproduction related | | |
| Motile sperm domain-containing protein 2 | Cp_Uni_012929; Cp_Uni_019096 | 3731; 4078 |
| Oocyte zinc finger protein | Cp_Uni_054497 | 590 |
| Sperm flagellar protein 1 | Cp_Uni_199947; Cp_Uni_199949; Cp_Uni_009524; Cp_Uni_199946; Cp_Uni_009523; Cp_Uni_009257; Cp_Uni_199950; Cp_Uni_146491; Cp_Uni_146495; Cp_Uni_146492; Cp_Uni_146494; Cp_Uni_146493; Cp_Uni_146496; Cp_Uni_258195; Cp_Uni_199951; Cp_Uni_199948;  Cp_Uni_258193; Cp_Uni_258194 | 3075; 3097; 1310; 1507;  1301; 5903; 2561; 1259; 1235; 1223; 1199; 777; 1187; 2100; 528; 3111; 856; 838 |
| Sperm flagellar protein 2 | Cp_Uni_258193; Cp_Uni_258194 | 856; 838 |
| Sperm motility kinase | Cp_Uni_113317; Cp_Uni_105298 | 2097; 4341 |
| Nuclear autoantigenic sperm protein | Cp_Uni_022427 | 1792 |
| Vitelline envelope sperm lysin receptor | Cp_Uni_114551 | 230 |
| Spermatogenesis-associated protein 1 | Cp_Uni_17123; Cp_Uni_171300; Cp_Uni_171308; Cp_Uni_171294; Cp_Uni_171306; Cp_Uni_171307; Cp_Uni_171280; Cp_Uni_171286; Cp_Uni_171303; | 3293; 3242; 3215; 3140; 3071; 2993; 2891; 3044; 2966; |
| Spermatogenesis-associated protein 7 | Cp_Uni_186358 | 2864 |
| Spermatogenesis-associated protein 4 | Cp_Uni_226086; Cp_Uni_226087 | 2650; 2631; |
| Spermatogenesis-associated protein 6 | Cp_Uni_158029; Cp_Uni_158023; Cp_Uni_158022; Cp_Uni_158028; Cp_Uni_158026 | 2438; 2452; 2410; 2386; 2344 |
| Spermatogenesis-associated protein 5 | Cp_Uni_027692; Cp_Uni_201263 | 2655; 1583 |
| Prostatic spermine-binding protein-like | Cp_Uni_243779; Cp_Uni_079128 | 611; 241 |
| Sperm-associated antigen 17-like | Cp_Uni_184717; Cp_Uni_184727; Cp_Uni_184716; Cp_Uni_184718;  Cp_Uni_184719; Cp_Uni_184723; Cp_Uni_184721; Cp_Uni_184722; Cp_Uni_027630; Cp_Uni_184720; Cp_Uni_184726 | 8879; 8935; 8791; 8735; 8855; 8911; 8767; 8711; 6371; 7204; 7148 |
| Spermidine synthase-like | Cp_Uni_257590; Cp_Uni_257595; Cp_Uni_257601; Cp_Uni_257604; Cp_Uni_013162; Cp_Uni_210472; Cp_Uni_210473; Cp_Uni_210474; Cp_Uni_210475 | 1832; 1733; 1705; 1672; 2737; 663; 757; 594; 1233 |
| Spermine oxidase-like | Cp_Uni_027816 | 3700 |
| Testis beta tubulin | Cp_Uni_246058 | 539 |
| Vitellogenin | Cp_Uni_091945; Cp_Uni_362950; Cp_Uni_248742; Cp_Uni_024626; Cp_Uni_064837; Cp_Uni_126430; Cp_Uni_219658; Cp_Uni_197581; Cp_Uni_044443; Cp_Uni_054115; Cp_Uni_372011; Cp_Uni_097211; Cp_Uni_134578; Cp_Uni_182033; Cp_Uni_153648; Cp_Uni_144055; Cp_Uni_334164; Cp_Uni_366163 | 7807; 284; 1005; 1228; 1046; 464; 878; 814; 848; 703; 657; 321; 292; 290; 671; 236; 250; 235 |
| Vitelline envelope zona pellucida domain 14 | Cp_Uni_135173; Cp_Uni_078868; Cp_Uni_317597; Cp_Uni_219154; Cp_Uni_353750 | 272; 650; 244; 226; 261 |
| Ovary development-related protein | Cp_Uni_040133; Cp_Uni_094758; Cp_Uni_277820; Cp_Uni_127348 | 444, 376, 554; 263 |
